# D-optimal design model and biosynthetic pathway for gentamicin production by *Micromonospora purpureochromogenes* NRRL B-16094

**DOI:** 10.1186/s12866-025-04001-8

**Published:** 2025-05-20

**Authors:** Muath Suliman, Amr S. Bishr, Sally T. K. Tohamy, Mohammad Y. Alshahrani, Khaled M. Aboshanab

**Affiliations:** 1 Department of Clinical Laboratory Sciences, College of Applied Medical Sciences, King Khalid University, Abha 9088, P.O. Box 61413 Saudi Arabia; 2 Department of Microbiology and Immunology, Faculty of Pharmacy , Ain Shams University, Cairo, 11566 Egypt; 3 Department of Microbiology and Immunology, Faculty of Pharmacy (girls), Al-Azhar University, 11651, Cairo, Egypt; 4 Central Labs, King Khalid University, AlQura’a,P.O. Box 960, Abha, 9088 Saudi Arabia

**Keywords:** Gentamicin, Kanamycin, Fortimicin, D-optimal design *Micromonospora purpureochromogenes* NRRL B-16094

## Abstract

**Background:**

*Micromonospora purpureochromogenes* NRRL B-16094, a natural producer of gentamicin (GEN), a 5,6-diglycosylated 2-dexoystreptamine-aminoglycoside antibiotic (2DOS-AGA) broad-spectrum bactericidal activity. In literature, limited studies are concerned with the biosynthetic route and various cultural conditions influencing GEN production.

**Methods:**

Therefore, this study aimed to explore the GEN biosynthesis pathway and compare it to that of fortimicin and kanamycin. In addition, four key environmental conditions influencing GEN production were statistically optimized using response surface D-optimal design (DOD). Herein, the biosynthetic pathway of GEN was proposed based on the biochemistry of the identified genes/proteins within the gene cluster. Comparing the GEN-biosynthetic gene cluster to that of kanamycin and fortimicin suggested that gentamicin biosynthesis could have originated from a combination of biosynthetic pathways of both antibiotics.

**Results:**

For the optimization experiments, culture media 4 (CM4) and 6 (CM6) gave the highest specific productivity at 6.36 and 3.80 µg/mg, respectively. A DOD quadratic model was successfully generated to optimize four key environmental factors. Predicted and experimentally confirmed optimized factors were an initial pH of 7, an incubation temperature of 30˚C, and an agitation of 300 rpm for 10 days. This resulted in a 13.5-fold increase (289.5 µg/mL) over that produced by the basic CM1 production medium (21.4 µg/mL) and 2.4 times (over that obtained by CM4 (123.7 µg/mL) as verified by HPLC analysis.

**Conclusion:**

DOD is an efficient tool for optimizing GEN. Accordingly, the optimized conditions are highly advisable during the scaling up of GEN production by *M. purpureochromogenes* NRRL B-16094.

**Supplementary Information:**

The online version contains supplementary material available at 10.1186/s12866-025-04001-8.

## Background

Aminoglycoside antibiotics (AGAs) are, with very few exceptions, products of higher differentiating actinomycetes. The kanamycins (KANs) and gentamicins (GENs) are members of the most relevant aminoglycoside subclass of the 2-deoxystreptamine aminoglycoside antibiotics (2DOS AGAs) [1–3]. Gentamicins and fortimicins (FTMs) are products of *Micromonospora* sp. while kanamycins are products of *Streptomyces* sp. On the other hand, GENs and KANs share the key intermediate, the paromomine during their biosynthesis however, FTMs have a fortamine intermediate [3, 4]. In the biosynthetic pathway, GENs were found to share some biosynthetic steps with both fortamine (such as FTMs) and 2-deoxyfortamine-containing AGAs (such as istamycins, ISMs) which contain a different type of aglycone [1, 4, 5]. The biosynthetic gene clusters of the three AGAs have been fully sequenced and deposited in the NCBI GenBank database [1]. Therefore, exploring the biosynthetic route of the respective AGAs will help researchers make certain modifications of the respective biosynthetic pathways which is known as combinatorial biosynthesis to get new members of these antibiotics exerting more potent bactericidal activities, particularly against multidrug-resistant pathogens [3].

GENs have been applied in various sectors, mostly to treat immunocompromised patients’ severe hospital-acquired infections, particularly those caused by MDR Gram-negative and Gram-positive pathogens [6, 7]. Several biosynthetic feeding studies were performed using certain isotope-labeled precursors to confirm the biosynthetic routes of both GENs and FTMs [8, 9]. In their biosynthetic pathways, the two KANs and GENs share the pseudodisaccharide paromamine. However, KANs are different in their chemical structures from GENs by some extra alterations that GENs share with the FTMs [1, 3, 10]. As a result, both KANs and GENs undergo 3”-amination in the neutral sugar unit that is joined to the 2-DOS aminocyclitol’s 6-hydroxyl group in the subsequent phases of modification. Conversely, the GENs and FTMs have similar 3’,4’-didehydroxylations and 6’-C-methylations [1, 7].

Through the optimization of numerous interrelating environmental factors impacting antibiotic production, computer-aided tools employing response surface methodology (RSM) are utilized to achieve maximal production of the target bioactive metabolites [11]. Using up to five levels for each variable with a minimal probability of missing data, the D-optimal design (DOD) is the most common in RSM experiments [12–14]. Recently, RSM was used to optimize cultural conditions influencing the production of ISMs, resulting in a 31-fold rise as compared to un-optimized factors [15]. In this study, the proposed biosynthetic pathway of GENs was illustrated in terms of the identified genes/proteins being involved in the biosynthesis and compared to those involved in related AGAs such as FTMs and KANs. In addition, various environmental and cultural media compositions previously known to influence AGA production were optimized using DOD.

## Materials and methods

### Microorganisms

*Micromonospora purpureochromogenes* NRRL B-16,094 (GENs producer) was provided by the USA-ARS Culture Collection (NRRL), Peoria, IL, USA. It was cultured in YMG (M65) broth (DSMZ, Braunschweig, Germany) incubated at 28˚C for 5 days, and stored as a lyophilized dried heavy suspension in bovine serum as recommended by the supplier. The standard *Bacillus subtilis* DSM 618 was used to calculate the antibacterial activity of the produced GENs [16].

### Identification of the GEM biosynthetic gene cluster

The GENs biosynthetic gene cluster was sequenced, assembled, and annotated as submitted into the NCBI GenBank database under the accession code, AJ628149.4 (https://www.ncbi.nlm.nih.gov/nuccore/AJ628149.4) [1]. FramePlot 2.3.2 software was used to determine the biosynthetic open reading frames (ORFs), where BLASTn and BLASTp were used to analyze the nucleotide and amino acid percentage identity, respectively. The ORFs were annotated using FramePlot 2.3.2 https://nocardia.nih.go.jp/frameplot/ [17]. BLAST https://blast.ncbi.nlm.nih.gov/Blast.cgi (accessed in October 2024) was used to analyze its associated ORF functions [18]. The pDRAW32 program, which may be downloaded for free, was used to construct and illustrate the corresponding ORFs: https://www.acaclone.com/(accessed October 25, 2024).

### The GEN biosynthetic pathway

Based on the biosynthetic biochemical role of each gene or protein that has been previously examined and validated in the literature, as well as the percentage of similarity to proteins or enzymes that are known to catalyze comparable biosynthetic roles, the biosynthetic pathway of GEN was proposed and characterized [5]. To shed light on the genetics and the processes involved in the formation of GENs, an effort was prepared to point out and examine the associated gene clusters. The foundation of this method was the understanding that all the genes involved in the synthesis of an antibiotic in Actinobacteria are usually contained in a single gene cluster. Furthermore, the majority of cases may be analyzed using partial sequence information obtained from the database because resistance and a few GEN biosynthesis genes have already been reported from our lab and others.

### The gentamicin (GEN) versus Kanamycin (KAN) and Fortimicin (FTM) biosynthetic pathways

The GEN, KAN, and FTM biosynthetic gene clusters have been sequenced, deposited to the NCBI GenBank database under the accession codes AJ628149, AJ628422, and AJ628421, respectively. The open reading frames (ORFs) present in each gene cluster have been detected and analyzed in terms of the function of each ORF in the biosynthetic pathways as well as the % identity and degree of conservation and location of the DNA segment harboring the respective antibiotic gene clusters. FramePlot 2.3.2 software [17], BLASTn and BLASTp [18], Pairwise sequence alignment(https://www.ebi.ac.uk/jdispatcher/psa) were used in the analysis.

## Optimization of GEN production

### Culture media

Trypticase soy broth (TSB broth) [19] as the basic culture medium was initially used to produce GEN by *M. purpureochromogenes* NRRL B-16,094, as was previously documented [20]. However, as shown in Table S1, an additional six different *Streptomyces*/*Micromonospora* culture mediums that were previously used for aminoglycoside production were examined to maximize GEN production [15]. The compositions of the tested culture media are displayed in Table S1.

### Evaluation of GEN production and cell growth

About 200 µL of the culture supernatant of the tested production medium was centrifuged and sterilized by membrane filtration after incubation (at the specified time of each experiment) as previously reported [20]. This was done in order to measure the antibacterial activity of the culture-free supernatant and determine whether GEN was produced. The resultant culture-free supernatant (150 µL) was assessed against *Bacillus subtilis* DSM 618 as formerly mentioned [21]. The amount of GEN produced by each culture was measured by measuring the inhibition zone in millimeters (IZ) after a 24-hour incubation period at 37˚C. A reference calibration curve was created using the standard GEN at different concentrations (µg/mL) by plotting known concentrations (represented as log concentration) of the standard GEN (CAS No. 1405-41-0, Merck, Darmstadt, Germany) against average inhibitory zone widths. The culture growth was calculated using the culture dry weight, as was formerly mentioned [15, 20, 22]. The specific productivity in µg/mg was calculated by dividing the observed GEN concentration (µg/mL) by the corresponding dry cell weight (mg/mL) [15].

### HPLC analysis for measuring the produced gentamicin

The quantity of GEN generated in the culture supernatant of *M. purpureochromogenes* NRRL B-16,094 was measured by HPLC analysis. Standard gentamicin (GEN; CAS No. 1405-41-0, Merck, Darmstadt, Germany) solutions at varying concentrations (20–3560 µg/mL) were produced in the employed mobile phase to create the standard curve. The Zorbax ODS (25 cm x 4.6 mm I.D.) chromatographic column (DuPont, Wilmgton, DE, USA) equipped with a C18 (3 cm x 4.6 mm I.D.) was used for the analysis, which was conducted using an HPLC Shimadzu CBM 40 at 30 °C with a UV detector set at 260 nm. For 60 min, 10 µL of a mobile phase consisting of a 1:1 mixture of HPLC-grade acetonitrile and methanol was injected at a flow rate of 1 mL/min. As previously mentioned [23], derivatization was performed using 2,4 dinitrofluorobenzene (DNFB) of 98% purity (Rochester, NY, USA).

### Culture media affecting GEN production

A 250 mL baffled Erlenmeyer flask was filled with 25 mL (TSB) of *M. purpureochromogenes* NRRL B-16,094. The mixture was then incubated at 28 °C at 200 rpm to produce seed culture. About 5% v/v of this seed culture (0.5 × 10^7^) was inoculated after three days of incubation with a total of 25 mL of each of the tested culture media (Table S1), which had been prepared in a 250 mL baffled Erlenmeyer flask and incubated at 28˚C for six days [20]at a shaking incubator (200 rpm) [19]. As previously reported, 200 µL was taken at the end of the 6-day incubation period to measure the amounts of GEN generated and the dry cell weight [15].

### Statistical optimization

In these studies, the optimal culture medium and incubation duration were selected from previous trials. For statistical optimization, The D-optimal design (DOD) of the Response Surface Method (RSM) was utilized using the statistical software application Design Expert v.7 [14, 24, 25]. The DOD was used in a quadratic design model to assess four factors: intial pH (coded as variable A), incubation temperature (coded as variable B), and agitation rate (coded as variable C) and incubation time (factor D). A set of 23 runs is produced by the DOD, as seen in Table 1. The analysis of variance (ANOVA) for the suggested model was used to acquire the statistical analysis’s findings [26].

**Table 1:** Different runs and the tested variable generated by DOD design

Run Order	pH (factor A)	Temperature ˚C (factor B)	Agitation rpm (factor C)	Incubation time (days)
1	7.6	29	115	10
2	9	37	300	6.5
3	5	37	100	6.0
4	7	37	190	7.5
5	9	25	300	10
6	9	25	300	10
7	9	37	160	3.0
8	9	25	300	3.0
9	7.5	32	295	10
10	5	25	100	3.0
11	9	25	300	3.0
12	9	25	100	6.0
13	7	31	300	5.0
14	5	25	100	10
15	9	37	100	10
16	5	37	300	3.0
17	5	37	300	3.0
18	5	37	300	10
19	5	30	200	6.5
20	7	25	200	6.5
21	5	25	240	3.0
22	5	25	300	7.5
23	7	32	100	3.0
**Factor**	**Name**	**Level (-1)**	**level (+ 1)**	
A	Initial pH	5	9	
B	Incubation temperature (B, ˚C)	25	37	
C	Agitation (C, rpm)	100	350	
D	Incubation Time (Days)	3	10	

### Experimental confirmation of statistical optimization

As stated in the approach, the unoptimized condition was compared to the ideal circumstances generated by the DOD in the lab based on the model’s significance (determined using ANOVA within the Design expert application).

### Statistical analysis

The mean and standard deviation were calculated using Microsoft 365’s Excel software, and each lab experiment was run in triplicate. The statistical analysis for the suggested model was determined using ANOVA [26].

## Results

### GEN biosynthetic gene cluster versus FTM and KAN gene clusters

**Fig. 1:**
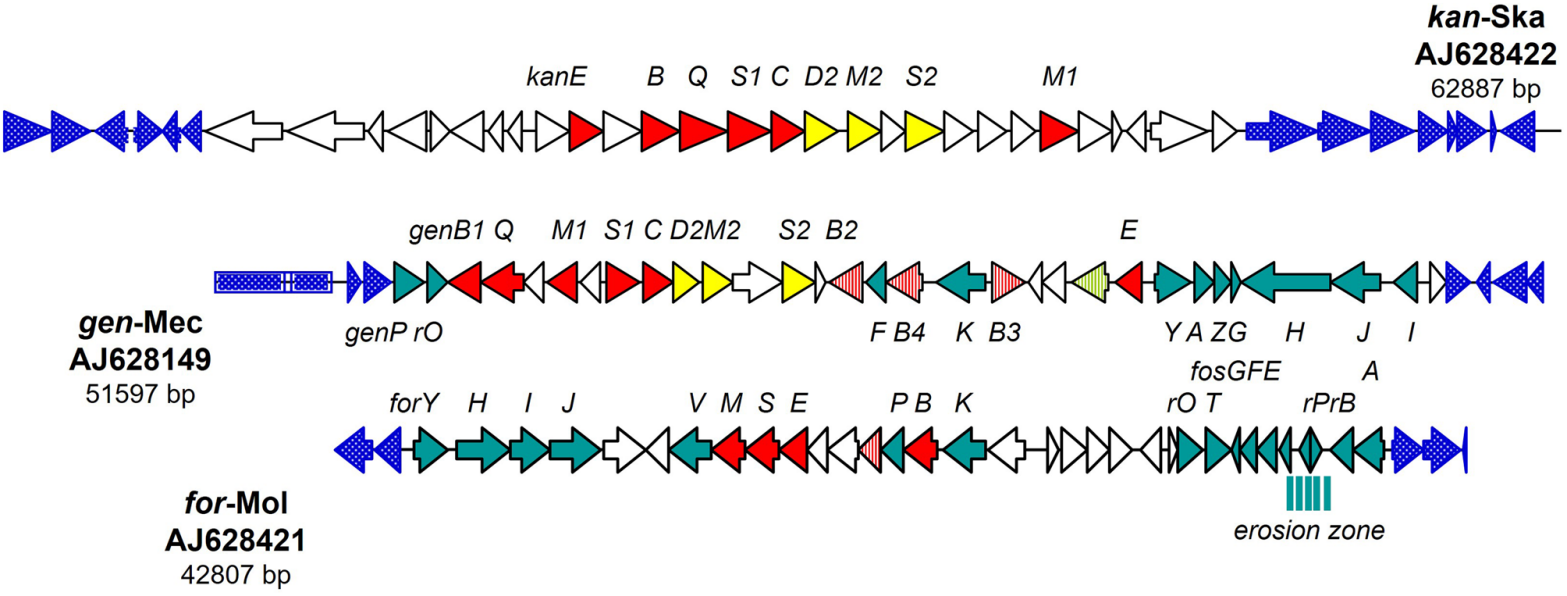
Biosynthetic gene cluster of gentamicin (GEN) produced by *M. purpureochromogenes* NRRL B-16,094 compared to that of fortimicin and kanamycin gene clusters. Open reading frames (ORFs) that are conserved in gentamicin (*gen*), kanamycin (*kan*), and fortimicin (*for*) biosynthetic gene clusters are colored red. ORFs conserved in *gen-* and *kan-* clusters are colored in yellow. ORFs conserved in *gen* and *for* clusters are colored in green. ORFs reside outside the ACAGA gene clusters (dotted blue)

The GEN, KAN, and FTM biosynthetic gene clusters have been sequenced, assembled, annotated, and submitted to the NCBI GenBank database under the accession codes, AJ628149, AJ628422, and AJ628421, respectively. The open reading frames (ORFs) present in each gene cluster have been detected and analyzed as depicted in Fig. 1. The following succinctly describes the cluster structures’ immediate interpretation for the three clusters under consideration: (i) comparing the three clusters reveals a surprisingly low overall degree of conservation; (ii) the gene order inside the clusters is likewise not generally conserved, for example, few gene subsets exhibit a somewhat preserved order, such as the gene set *for*HIJ corresponding to *gen*HJ or the subcluster *kan*S1CD2M2DS2 related to *gen*S1CD2M2D1S2. The alignment of the DNA segment coded for the *for*HIJ from the fortimicin biosynthetic gene cluster (NCBI accession code, AJ628421) with the DNA segment coded for *gen*HJ from the gentamicin biosynthetic gene cluster (NCBI accession code, AJ628149) showed almost 95.0% conservation of the nucleotide sequence is displayed in Fig. S1. In addition, the alignment of the DNA segment coded for the *kan*S1D2M2D1S2 from the kanamycin biosynthetic gene cluster (NCBI accession code, AJ628422) with the DNA segment coded for *gen*S1D2M2D1S2 from the gentamicin biosynthetic gene cluster (NCBI accession code, AJ628149) about 97% conservation of the nucleotide sequence as depicted in Fig. S2. (iii) the adjacent genomic regions are not preserved at all, but they are highly conserved in the full-genome sequenced *S. coelicolor* A3(2).

**Fig. 2:**
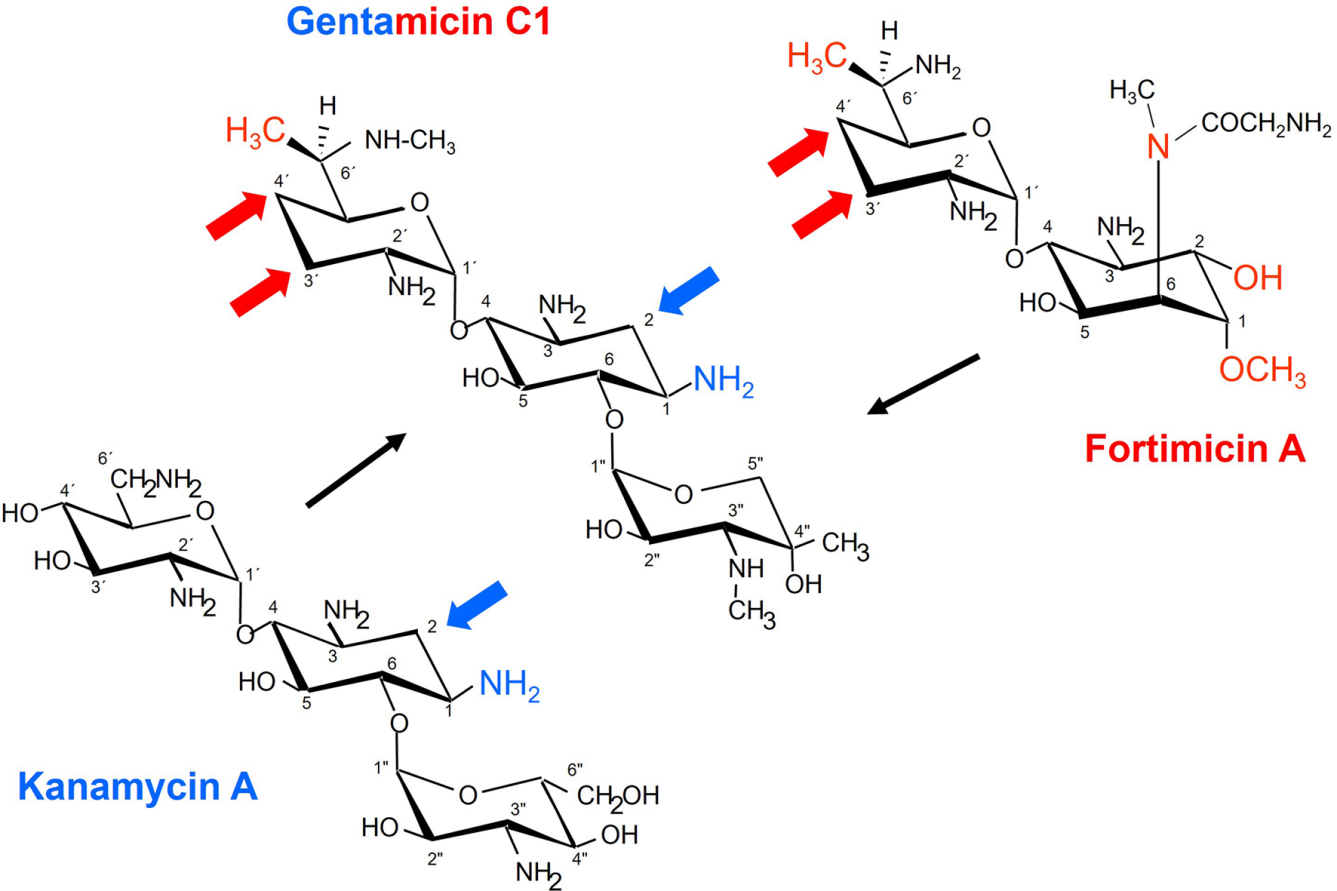
Chemical structure of gentamicin, fortimicin and kanamycin. Arrows indicate similarities in structure in both sugar and aglycone moieties among three aminoglycoside antibiotics (AGAs)

With the exception of the *gen*B1/*for*B and *gen*Q/*for*D2 encoded pairs of identical proteins, most of the gene/protein families are conserved in the gene clusters for the majority of the 2DOS-AGAs, which form a paromamine precursor in their biosynthetic routes. The following genes were covered by the pairs of strongly conserved segments between the *kan*- and *gen*-clusters: *kan*E/*gen*E, *kan*S2/*gen*S2, *kmr*, kanM1/*gmr*A, and *gen*M1. The following sets of equivalent gene sets were also covered by the DNA segments of conservation between the *for*- and *gen*-clusters: *for*HIJ/*gen*HJ, *for*D2/*gen*Q, *for*PBK/*gen*KB3PB4 (genes B3 and B4 appear to have originated from a recent gene duplication), *for*T/genI, *fos*DEFG/*gen*YAFG, *fos*C/*gen*W, *fmr*B,*fos*A/*gmr*B, and *gen*P, as illustrated in Fig. 1. These findings suggest that GEN biosynthesis could originate from a combination of FTM and KAN biosynthetic pathways, and this is evidenced by the resemblances in the modified sugars and aglycone moieties of the respective AGAs as depicted in Fig. 2.

### Biosynthetic pathway of GEN

**Fig. 3:**
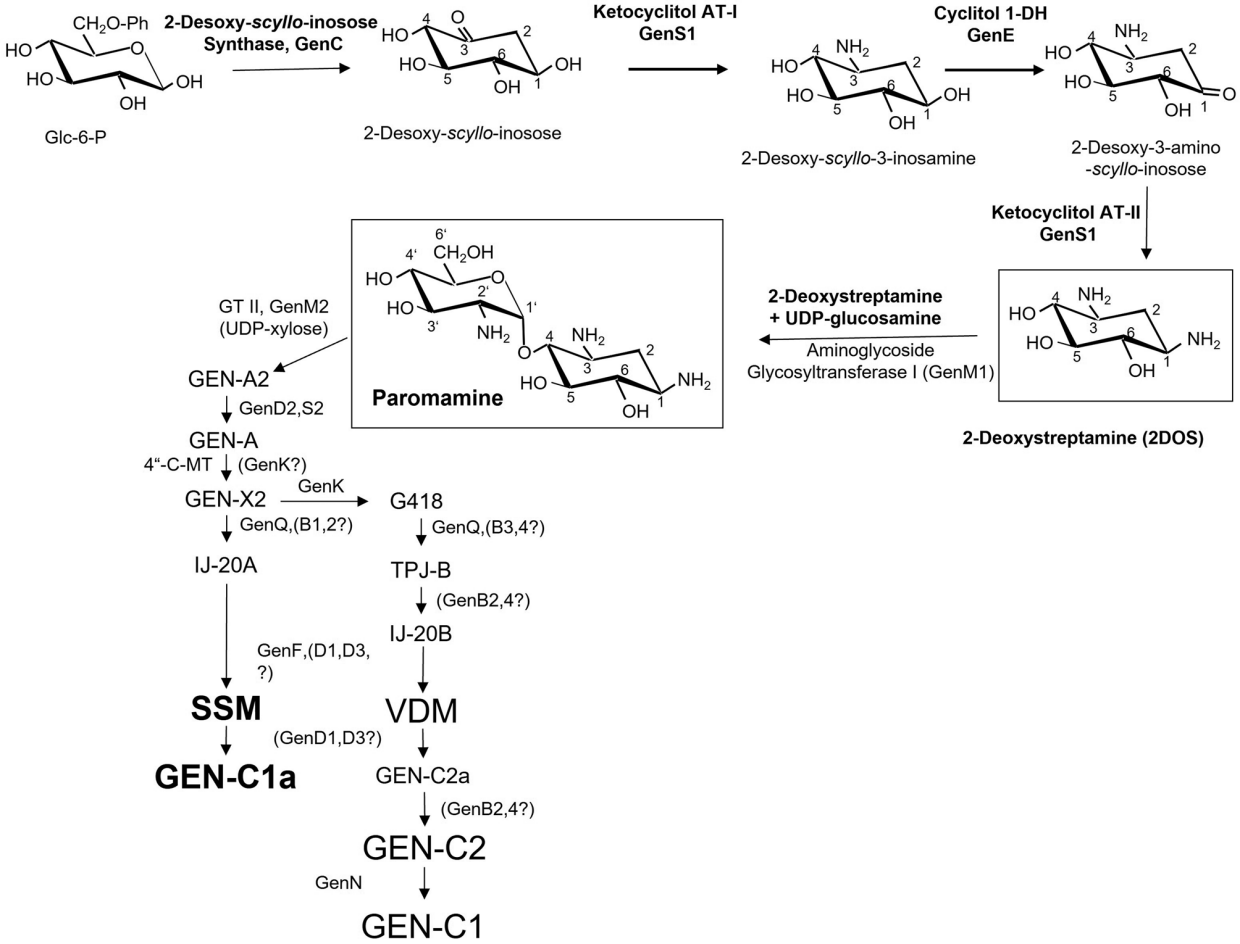
The proposed biosynthetic pathway of gentamicin in its natural producer, *M. purpureochromogenes* NRRL B-16,094. AT, aminotransferase, GT, glycosyltransferase, GEN, Gentamicin, GEN-C (Gentamicin C-complex), SSM, Sisomycin, VDM, Verdamicin

Based on the previously biochemically identified genes and proteins involved in 2-deoxystreptamine (2DOS), basic cyclitol, and specific glycosylation or sugar modification processes, the putative GEN biosynthesis route was outlined in Fig. 3. Based on amino acid sequence and structural resemblance to homologous proteins or related ACAGAs that participate in comparable enzymatic activities, the other genes and proteins that are present in the GEN biosynthesis gene cluster but have not yet been biochemically identified were added to the pathway. However, as illustrated in Fig. 1, the GEN biosynthetic route theory is based on an examination of the gene products that the *gen-*cluster encodes.

### Production optimization of gentamicin (GEN)

#### Assessment of GEN concentration

A standard curve was created by plotting the GEN concentrations against the Area Under the Curve (AUC) (Fig. S3). The GEN- was computed using the suggested linear equation. The formula for a concentration was y = 0.205 x + 67.86. x is the GEN concentration in µg/mL, and y is the AUC with R2 = 0.9925 (Fig. S3). GEN concentration was also calculated from the following equation: Y (inhibition zone diameter (mm) = 17.467X -2.9147 where X is antilog GEN concentration in µg/mL with R^2^ = 0.939 as shown in Fig. S4.

### Effect of culture media

Table 2 displays the dry cell weight and GEN generated by *M. purpureochromogenes* NRRL B-16,094. The highest specific productivity was found in CM4 and CM6, with respective values of 6.36 and 3.8 µg/mg and corresponding GEN productivity of 103.9 and 66.23 µg/mL. As a result, CM4 was selected as the ideal culture medium for the next examinations.

**Table 2:** Corresponding GEN concentration produced by *Micromonospora purpureochromogenes* NRRL B-16,094 and specific productivity obtained from the tested culture media

Nr.	average IZ	antiLog	^a^Corresponding GEN concentration (µg/mL)	Dry cell weight (mg/mL)	^b^specific productivity (µg/mg)
CM1 (basic culture medium)	20.33	1.330778	21.4	15.33	1.37
CM2	13.33	1.144635	13.95	10.66	1.31
CM3	18.66	1.45328	28.39	13.66	2.07
CM4	28.33	2.013243	103.9	16.33	6.36
CM5	14.66	1.221652	16.59	12.33	1.34
CM6	25.66	2.09026	66.23	17.33	3.8
CM7	22.33	1.6658	45.7	15.33	2.49

GEN, Gentamicin; IZ, inhibition zone (mm); SD, standard deviation. The composition of the seven culture media (CM1-CM7) is outlined in Table S1 (supplementary data)

### D-Optimal design (DOD)

Using the DOD, four parameters—initial pH, incubation temperature (˚C), agitation rate (rpm), and incubation period (days)—have been optimized. Both the predicted and observed responses for the 23 runs have been documented, as seen in Table 3. According to ANOVA analysis, the model’s F-value was 11.21, meaning it is significant (Table 4). ANOVA study confirmed the applicability of the model and showed that the generation of GEN was significantly influenced by the initial pH (factor A), agitation (factor c), and incubation duration (factor D). Additionally, CD and A2 were significant (P value < 0.05), according to the data. However, the measured incubation temperature range of 25–37 ˚C showed no discernible effect on GEN production in terms of the recorded inhibition zone, (P value, 0.3001). Eventually, the equation generated by DOD for evaluation of the amount of GEN produced is:

**Table 3:** Runs of the D-optimal design for optimizing GEN production showing observed and predicted responses

Run Order	pH (factor A)	Temperature ˚C (factor B)	Agitation rpm (factor C)	Incubation time days (factor D	Observed response	Predicted response
1	7.6	29	115	10	27	28.05
2	9	37	300	6.5	20	20.45
3	5	37	100	6.0	16	17.99
4	7	37	190	7.5	26	24.9
5	9	25	300	10	19	19.66
6	9	25	300	10	20	19.66
7	9	37	160	3.0	12	12.27
8	9	25	300	3.0	22	19.98
9	7.5	32	295	10	26	27.10
10	5	25	100	3.0	12	11.75
11	9	25	300	3.0	18	19.98
12	9	25	100	6.0	17	13.69
13	7	31	300	5.0	26	28.07
14	5	25	100	10	26	25.21
15	9	37	100	10	20	21.81
16	5	37	300	3.0	27	24.28
17	5	37	300	3.0	27	27.28
18	5	37	300	10	26	23.97
19	5	30	200	6.5	22	21.16
20	7	25	200	6.5	30	25.06
21	5	25	240	3.0	14	17.7
22	5	25	300	7.5	22	23.45
23	7	32	100	3.0	16	16.31
*Factor*	*Name*	*Level (-1)*	*level (+ 1)*			
A	Initial pH	5	9			
B	Incubation temperature (B, ˚C)	25	37			
C	Agitation (C, rpm)	100	350			
D	Incubation Time (Days)	3	10			

Final Equation: Inhibition Zone (GEN Produced) = -67.25875 + (20.18893 * Initial pH) + (0.052546 *Incubation temperature) + (0.088 * Agitation rate) + (2.83075 * Incubation Time)– (9.58709E-003 * Agitation rate* Incubation Time)–(1.50768 * Initial pH^2^)

**Table 4:** ANOVA analysis of the GEN production using the D-optimal design quadratic model

Source	Sum of Squares	Df	Mean Square	F Value	*p*- value
Model	490.55	6	81.76	11.21	< 0.001 (significant)
A- Initial pH	55.40	1	55.40	7.60	0.014
B- Incubation temperature	1.72	1	1.72	0.24	0.633
C- Agitation	114.30	1	114.30	15.68	0.001
D. Incubation time	145.03	1	145.03	19.89	0.001
CD	139.76	1	139.76	19.17	0.005
A^2^	145.81	1	145.81	20.00	0.004
Residual	116.67	16	7.29		
Lack of Fit	108.07	13	8.32	2.54	0.203 (Not significant)
Pure Error	8.50	3	2.83		
Cor Total	607.00	22			
Stander deviation (SD)	2.70		R-Squared	0.807	
Mean	21.35		Adj R-Squared	0.735	
C.V.%	12.65		Pred. R-Squared	0.640	
PRESS	216.55		Adeq Precision	10.972	

Lambda = 1 (no transformation is required)

GEN activity (IZ mm) = --67.25875 + (20.18893 * Initial pH) + (0.052546 *Incubation temperature) + (0.088 * Agitation rate) + (2.83075 * Incubation Time)– (9.58709E-003 * Agitation rate* Incubation Time)– (1.50768 * Initial pH^2^).

### Contour and 3D-dimensional plots

**Fig. 4:**
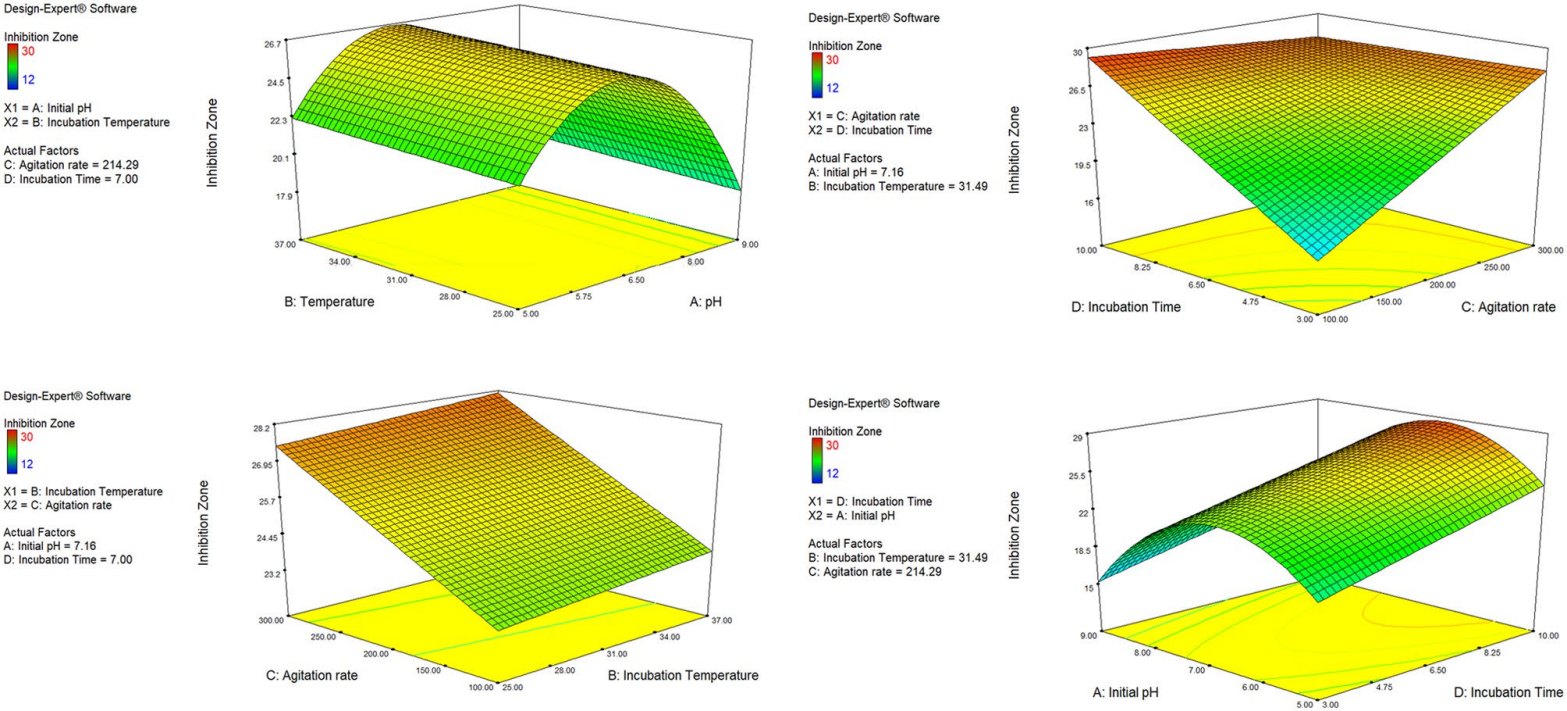
Three-dimensional (3D) surface plots for the effects of: **a**) initial pH versus incubation temperature; **b**) incubation temperature versus agitation; **c**) agitation versus incubation time; **d**) incubation time versus initial pH on gentamicin (GEN) produced by *M. purpureochromogenes* NRRL B-16094

The contour (Fig. S5-S8, supplementary file) and three-dimensional plots of the experienced four variables including, initial pH versus incubation temperature (Fig. 4a), Incubation temperature versus agitation rate (Fig. 4b), agitation rate versus incubation time (Fig. 4c), and incubation time versus initial pH (Fig. 4d) showed that the maximum GEN production of *M. purpureochromogenes* NRRL B-16,094 was attained at an initial pH of 7.0, an incubation temperature of 30˚C, an agitation rate of 300 rpm and incubation time 10 days using the production medium CM4.

### Validation of the generated model

**Fig. 5:**
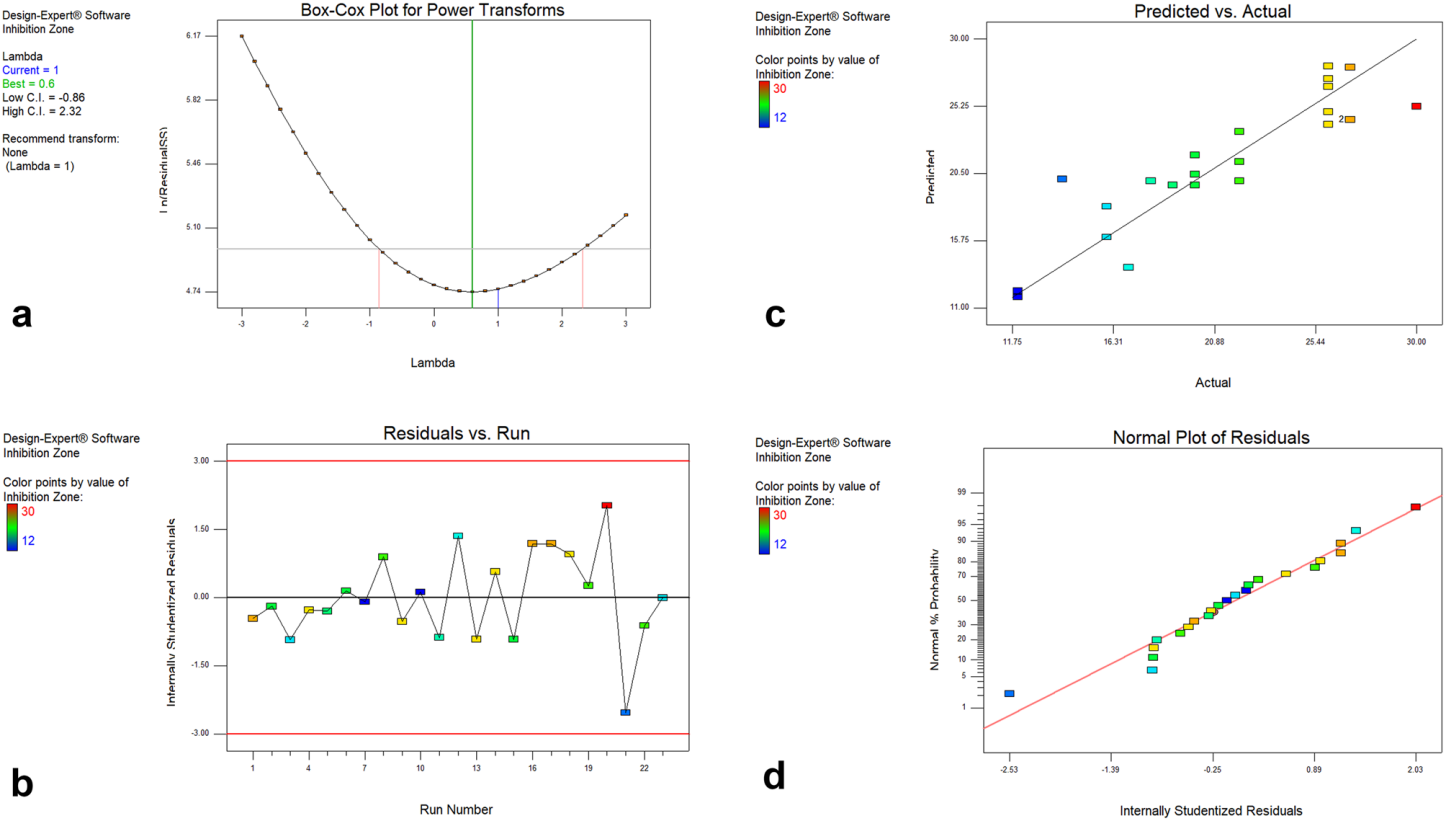
Model Diagnostics of the D-optimal design (DOD): (**a**) Box-Cox plot for Power Transforms; (**b**) Residuals vs. Run plot; **c**); Predicted vs. actual plot; **d**) Normal plot of Residuals on GEN produced by *M. purpureochromogenes* NRRL B-16094

As seen in Fig. 5a, box-cox plot analysis demonstrated that the model was applicable and significant (lambda current = 1 and the best = 0.6), and no further transformation was needed. Additionally, the residuals vs. run plot (Fig. 5b) showed that the model fits the data, and the plot showed that the actual values were very similar to the predicted ones (Fig. 5c). Additionally, the residuals’ normal plot, which was created by plotting the internally studentized residuals against normal percentage probabilities, demonstrated that the residuals had a normal distribution and that the points drew a straight line, as seen in Fig. 5d.

### Laboratory verification of the optimized factors generated by the DOD model

To achieve maximum GEN production as recommended by DOD design, the optimal levels of the four variables under test—initial pH 7.0, incubation temperature 30˚C, incubation time 10 days, and agitation rate 300 rpm—were selected. This resulted in an average inhibitory zone of 35 mm, proving the model’s validity. This amount was equivalent to 245.5 µg of GEN per milliliter. This led to a 2.4-fold increase in comparison to the optimized culture media (CM4, 7 days; 103.9 µg/mL) and an 11.5-fold rise in comparison to the unoptimized culture conditions (TSB broth for 6 days incubation; 21.4 µg/mL).

### Experimental confirmation using HPLC

**Fig. 6:**
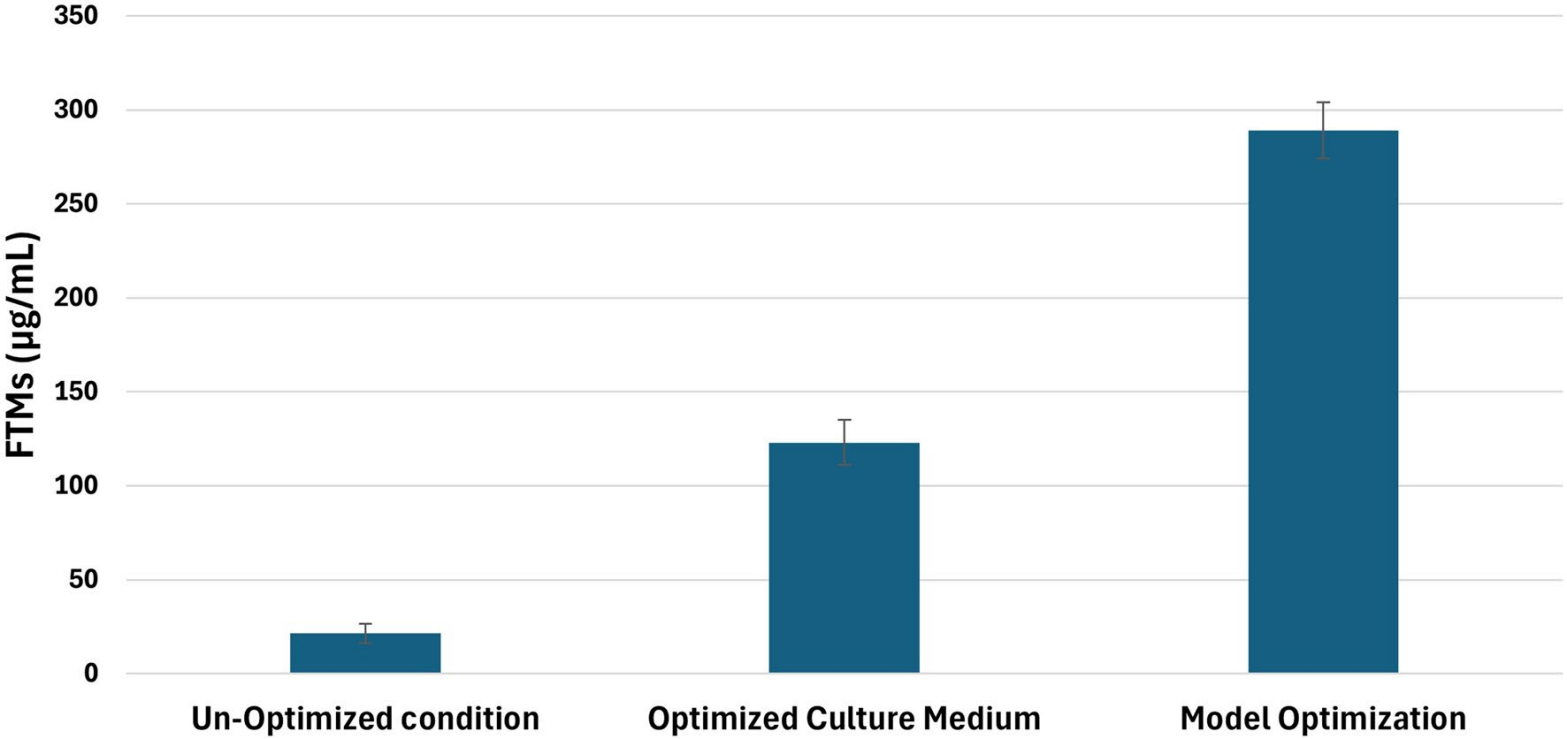
Graphical presentation of the GEN produced by *M. purpureochromogenes* NRRL B-16,094 under un-optimized, optimized culture medium (CM4) and optimized condition using the D-optimal model

Figure S7 shows the HPLC chromatogram of standard GEN and the one generated in the *M. purpureochromogenes* NRRL B-16,094 culture supernatant. Standard GEN’s retention time was 4.5 min. From 4.1 to 4.5 min, the generated GEN’s retention period in the culture supernatant was noted. HPLC analysis was used to verify the generated GEN, and the resulting AUC of the generated GEN was computed using the defined calibration curve as depicted in Fig. S9. As depicted in Fig. 6, the model produced 289.2 µg/mL (corresponding to AUC 1079) of GEN, whereas CM4 produced 123.7 µg/mL (equivalent to AUC 512.8), resulting in a roughly a 13.5-fold increase in GEN production as compared to basic production conditions (21.4 µg/mL, using CM1 production medium, incubation time 6 days, 28 ˚C incubation temperature and 200 rpm agitation).

## Discussion

The first objective of this study is to explore the proposed biosynthetic pathway of GEN and compare it to those of KAN and FTM antibiotics. The identified genes and proteins involved in the biosynthesis of GENs were used in this work to show the suggested biosynthetic route, and they were compared to those involved in related AGAs like FTMs and KANs. Starting from the expectation that the 2DOS aminoglycoside gentamicin resulted from a combination of gene sets for the production of KAN- and FTM-type, the working hypothesis that the GEN (*gen*) cluster should contain both strong and alternative relationships to both production gene clusters of the other two gene clusters (*kan*,* for*) was tested by isolation and sequence analysis of the three gene clusters and their immediate genomic vicinity. For this purpose, the genomic DNAs harboring the biosynthetic gene clusters of *S. kanamyceticus* DSM 40,500 (kanamycin-producing, AJ628422), *M. purpureochromogenes* NRRL B-16,094 (gentamicin-producing, AJ628149), and *M. olivasterospora* DSM 43,868 (fortimicin-producing, AJ628421), were compared and analyzed.

The explanation of the cluster structures for the three antibiotics is: (I) the overall degree of conservation, when all three clusters are compared, is unexpectedly low; (II) the gene order inside the clusters is also not conserved at large, i.e. only a few subsets of genes show a somewhat retained order, e.g. the subcluster *kan*S1CD2M2DS2 is related to *gen*S1CD2M2D1S2, or the gene set *for*HIJ corresponds to *gen*HJ; (III) the adjoining genomic regions are not conserved however, they are enriched with ORF’s that are highly conserved in the completely analyzed streptomycete genomes, *S. coelicolor* A3 [27] and *S. avermitilis* [28]. Interestingly, the genomic DNA sequence from *M. purpureochromogenes* NRRL B-16,094 (*M. echinospora* strain ATCC 15835) was obtained comprising 43 ORFs as depicted in the results. Two additional groups from *M. echinospora* strain ATCC 15,835 that have previously been submitted to the NCBI database (NCBI AY524043.1 and AJ575934.2) also studied a subsegment of the sequenced genomic area presented here. The sequences that overlap is nearly identical: The disclosed sequence comprises a portion of an rRNA operon that appears to surround the GEN-production cluster on one side. Because it shares the closest link with the *rrn*E operon of *S. coelicolor* A3(2) [27]. Our results allow us to hypothesize that the gen-cluster may have essentially resulted from a merger of the progenitor gene clusters *for* and *kan*.

The following is a summary of the findings: (I) The gen-cluster DNA displayed notable stretches of similarity to both other DNAs in mutually exclusive regions, whereas the *kan*- and *for*-cluster DNAs did not exhibit any notable similarities. In general, the two flanking segments of the *gen*-cluster contained segments of strong similarity towards the *for*-cluster, while the central part of the *gen*-cluster essentially contained the similarity regions towards the *kan*-cluster but was broken up by another stretch of for-similarity. This made the distribution of similar regions rather complex. (II) Numerous rearrangements, including insertions of non-conserved genomic material, deletions, inversions, and displacements, could be found within these sequences. (III) The following genes were covered by the pairs of highly conserved regions between the *gen*- and *kan*-clusters: *kan*E/*gen*E, *kan*S1CD2M2/*gen*S1CD2M2, *kan*S2/*gen*S2, *kmr*,* kanM*1/*gmr*A,*gen*M1. (IV) The *for*HIJ/genHJ, *for*D2/genQ, *for*PBK/*gen*KB3PB4, *for*T/genI, *fos*DEFG/*gen*YAFG, *fos*C/genW, *fmr*B,*fos*A/*gmr*B, and *gen*P were among the sets of analogous gene sets spanned by segments of conservation between the for- and gen-clusters. (V) According to the data on the gene clusters for the production of neomycin, paromomycin, ribostamycin, lividomycin, tobramycin, apramycin, and hygromycin B, the level of conservation between the gen/for pairs of equivalent genes/proteins is generally much higher (55 to 95%), and the majority of these genes do not occur in any other of the currently analyzed AGA gene clusters [1].

The immediate interpretation of the cluster structures for the three clusters under investigation is summarized as follows: The gene order within the clusters is also not generally conserved; for instance, only a small number of gene subsets show a somewhat preserved order, such as the gene set f*or*HIJ corresponding to *gen*HJ or the subcluster *kan*S1CD2M2DS2 related to *gen*S1CD2M2D1S2. Comparing the three clusters reveals a surprisingly low overall degree of conservation. The nucleotide sequence conservation of the *for*HIJ DNA segment from the fortimicin biosynthesis gene cluster (NCBI accession code: AJ628421) was nearly 95.0% when it was aligned with the genHJ DNA segment from the gentamicin biosynthesis gene cluster (NCBI accession code: AJ628149). Furthermore, as shown in the results (supplementary data), there is approximately 97% nucleotide sequence conservation when the DNA segments coding for *gen*S1D2M2D1S2 from the gentamicin biosynthesis gene cluster (NCBI accession code, AJ628149) and *kan*S1D2M2D1S2 from the kanamycin biosynthesis gene cluster (NCBI accession code, AJ628422) are aligned. The obtained data support our hypothesis that gentamicin biosynthesis could have originated from a combination of biosynthetic pathways of kanamycin and fortimicin antibiotics.

The second objective of this study is to statistically optimize GEN production by its natural producer, *M. purpureochromogenes* NRRL B-16094. Accordingly, in this study, the production of GEN was evaluated using various actinomyces/antibiotic production media, and the results showed that two media showed maximum specific productivity, namely CM4 which is also named M65 (DSMZ, Braunschweig, Germany), followed by CM6 (also named as APM; [29], respectively. Therefore, the CM4 culture medium was selected for the next experiments. Our results are in accordance with previous studies where the respective AGA production media gave maximum specific productivity of paromomycin [20], istmaycin [15], and fortimicin [30]. As previously reported, environmental culture conditions, for instance, culture media compositions, incubation temperature, agitation rate, initial pH, and incubation time, play an important role in the production of antibiotics by their natural producers [31, 32]. Therefore, in this study, four environmental factors, including the initial pH of the selected production medium (CM4), incubation temperature, agitation rate, and incubation time, have been evaluated and statistically optimized for maximum GEN production. Accordingly, RSM using DOD was selected for accomplishing this purpose where the tested factors were as follows: initial pH (factor A), incubation temperature (factor B), agitation rate (factor C), and incubation time (factor D) each factor was tested at two levels (-1, and + 1). As previously stated, the response surface DOD was used for its appropriateness since four interacting components, each at two levels, must be assessed (mixed models with multi-response) [11, 25].

The D-optimal design was chosen for our model’s construction because it is characterized by having the best subsets of all possible experiments, is a practical model to work with harsh or extreme conditions, and is highly sensitive and predictive with the least percentage bias [24, 33]. The main goal of D-optimal design is to influence optimal system fulfillment by simultaneously improving a large number of elements [25]. It investigates how changes in factor levels affect a selected response and, in turn, how responses can be anticipated [25]. Our study’s DOD produced 23 experiments that, according to ANOVA, produced a significant model (*P value* < 0.001). With just a 0.01% probability that a “Model F-Value” may occur due to noise, the Model F-value of 11.21 indicates that the model is significant [34]. Furthermore, ANOVA analysis revealed that three of the four factors—the initial pH, agitation rate, and incubation time—had significant effects (p-value < 0.05) on the generation of GEN within each tested range, which was in line with other findings [35, 36]. However, within the tested range (25–37 ˚C) of the incubation temperature (factor B) does not significantly affect the formation of GEN (*p-value* = 0.633). We regard these results as new evidence that *M. purpureochromogenes* NRRL B-16,094 produces GEN. The Lack of Fit “F-value” for the DOD model was 2.54, which indicates non-significant behavior in relation to the pure error (p-value = 0.203). In essence, a non-significant lack of fit is considered a model’s confidence marker. The lab results showed a remarkable charge admiration with a low coefficient of variation (CV) of 12.65%. The CV is a valuable guarantee to ascertain the importance of accuracy in assessing the readings. The “Adj R-Squared” of 0.735 and the “Pred R-Squared” of 0.640 are reasonably coordinated. For the signal-to-noise ratio, “Adeq Precision” must be four or above. It’s interesting to note that our model’s ratio of 10.972 indicated an acceptable signal, and it can be used to explore the design space. The optimal values of 7 for the initial pH, 30 ˚C for the incubation temperature, 300 rpm for the agitation, and 10 days for the incubation time were determined by the contour and three-dimensional graphs of the factors of our model that were predicted. Our lab confirmed the expected values collectively, and the results showed a 2.4-fold increase over the optimized production medium (CM4) and a 13.5-fold rise over the unoptimized culture condition as verified by HPLC analysis using basic AGA production CM1 incubated at 28 ˚C, with an agitation of 200 rpm for 6 days.

## Conclusion

The biosynthetic pathway of GEN has been elucidated based on an analysis of the genes/proteins that the gentamicin *(gen-)* gene cluster encodes. By comparing the *gen-* biosynthetic gene cluster to those of kanamycin and fortimicin, results revealed that gentamicin biosynthesis could have originated from a combination of fortimicin and kanamycin biosynthetic pathways, which is evidenced by the presence of many structural similarities in the modified sugars and aglycone moieties. Six actinomyces/aminoglycoside production media were evaluated for maximum production of gentamicin and compared to the basic gentamicin production medium (TSB). Results showed that CM4 gave the highest specific productivity. The initial pH of the CM4 (pH ranged from 5 to 9), the incubation temperature (25–37 ˚C) and agitation rate (100–300 rpm), incubation time (3–10 days) were statistically optimized using a D-optimal design resulting in a 2.4-rise increase as compared to the optimized culture media and a 13.5-fold increase as compared to the unoptimized culture conditions. The resulting optimized conditions are highly recommended for scaling up the production of GEN produced by *M. purpureochromogenes* NRRL B-16,094, taking into consideration factors influencing the scale-up process such as mass transfer limitation (inoculum size), metabolic flow redistribution, constant power input per unit volume, mixing time and agitation rate in terms of stirrer speed and aeration rate as previously reported [37–39].

## Electronic supplementary material

Below is the link to the electronic supplementary material.

Supplementary Material 1

## Data Availability

All data generated or analyzed during this study are included in this published article and supplementary file. The gentamicin, kanamycin and fortimicin biosynthetic gene clusters were deposited in the NCBI GenBank database under the accession code, AJ628149 (https://www.ncbi.nlm.nih.gov/nuccore/AJ628149), AJ628422 (https://www.ncbi.nlm.nih.gov/nuccore/AJ628422), and AJ628421 (https://www.ncbi.nlm.nih.gov/nuccore/AJ628421), respectively.
