# Supplementary material for: D-optimal design model and biosynthetic pathway for gentamicin production by Micromonospora purpureochromogenes NRRL B-16094
Source: BMC Microbiol. 2025 May 20;25:309. doi: 10.1186/s12866-025-04001-8 (PMC12090562; doi:10.1186/s12866-025-04001-8)
Supplement: Supplementary file 1 — Supplementary Material 1 [file 12866_2025_4001_MOESM1_ESM.docx]

**Table S1.** composition of actinomycetes Culture media for optimizing the production of gentamicin (GEN)

| **Name/(code)** | **Medium composition** | **value** | **unit** | **Reference/Source** |
| --- | --- | --- | --- | --- |
| CM1 (basic GEN production medium) | Trypticase soy broth (Oxoid) or Soyabean casein digest broth | 30.0 | g/L | (Kieser et al. 2000) |
| CM2 | Sucrose | 103.0 | g/L | (Babcock and Kendrick 1988) |
|  | MgCl_2_ | 10.0 | g/L |  |
|  | Glucose | 5.0 | g/L |  |
|  | Yeast extract (Difco) | 5.0 | g/L |  |
|  | TES, pH 7.2 | 4.58 | g/L |  |
|  | After autoclaving add: |  |  |  |
|  | CaCl_2_ (5 M) | 2.0 | mL |  |
|  | Trace element solution | 2.0 | mL |  |
|  |  |  |  |  |
| CM3 | Soy meal | 20.0 | g/L | (Distler et al. 1985) |
|  | Mannit | 20.0 | g/L |  |
|  |  |  |  |  |
| CM4 | Glucose | 4.0 | g/L | (DSMZ, Braunschweig, Germany) |
|  | Yeast extract | 4.0 | g/L |  |
|  | Malt extract | 10.0 | g/L |  |
|  |  |  |  |  |
| CM5 | Yeast extract | 3.0 | g/L | (Kieser et al. 2000) |
|  | Peptone | 5.0 | g/L |  |
|  | Malt extract | 3.0 | g/L |  |
|  | Glucose | 10.0 | g/L |  |
|  | Sucrose | 340.0 | g/L |  |
|  | After autoclave add MgCl_2_ (1 M) | 10 | mL |  |
|  |  |  |  |  |
| CM6 | Soy meal | 30.0 | g/L |  |
|  | Ammonium chloride | 4.0 | g/L | (Nam and Ryu 1985) |
|  | Calcium carbonate | 5.0 | g/L |  |
|  | Glycerol | 40 | mL |  |
|  |  |  |  |  |
| CM7 | Soluble starch | 20 | g/L | (WILLIAMS et al. 1971) |
|  | KNO_3_ | 2 | g/L |  |
|  | K_2_HPO_4_ | 1 | g/L |  |
|  | MgSO_4_. 7H_2_O | 0.5 | g/L |  |
|  | NaCl | 0.5 | g/L |  |
|  | CaCO_3_ | 3 | g/L |  |
|  | Trace element solution | 2.0 | mL |  |

APM, Aminoglycoside Production Medium; SpMR, Protoplast Regeneration Medium, SMA, Soy Meal Mannit Agar, YEME, Yest Extract Malt extract medium; SNA, Starch nitrate agar.

**ForHIJ ---ATGA------CTCTCGACTTCCTGCGTGTTCGGAATCAACGTGTGCGCCA--GAAGT 49**

**GenHJ ATGAAGAATCTGCGATTAGCTTCCCTGGTCGAGCGCATGTCGCGTGGGAGCGCGCCGAGC 60**

*** ** * * * **** * ** * **** * ** ****

**ForHIJ GGGCCACGCTCGACGGCAC----------CCGCGACGGTGCCGCCCCGGAGGAGAACCTC 99**

**GenHJ GCGCTCGACCCGACGGAGCGGTCCCGGGCATCGCGTGGGACGAAGACCGAGATCGACTGG 120**

*** ** * ****** * ** * * *** ****

**ForHIJ GACGGGGTCAAGGACCCGGACTTCCTCGCGCTCGGCCTGGGCGCGACGAACATGATGGCA 159**

**GenHJ ACGGGTATCCACAATCCCGACTTCGTCGCACTCGGGCTGGGCGGGACCAGCATGATGGCG 180**

**** ** * * ** ****** **** ***** ******* *** * ***********

**ForHIJ ATGTTGTGGTCGCTGGCCCTCGGCCGGCGAGTGGTGGGCGTGGAGATGCGCGGCGACCCG 219**

**GenHJ ATGCTGTGGTCGGTGGCCAACGGTCGTCGCTGCGTCGGCGTCGAACTGCGTGGTGACCCC 240**

***** ******** ***** *** ** ** ** ***** ** **** ** *******

**ForHIJ TCGCTGGGCGTCCACTGGAACATCCGCGAGGACCTGTTCCACCACTGGGGCCTCATCGAC 279**

**GenHJ TCGCTCGGTGTCCACTGGAACATCCGTGAGGACTTCTGGCACCACCTCGGTCTGATCGAT 300**

******* ** ***************** ****** * * ****** ** ** *******

**ForHIJ AAGCTGATGCTGGAGCGTTACGGCGAGGAGAACCTCCCCCACCGCGGCGACGGCAAGTTG 339**

**GenHJ CAGCTCATGCTCGAGCGGTACGGCGAGGAGGGCATCCCCCGCAAGGGTGACGGCCGGCTC 360**

****** ***** ***** ************ * ****** * ** ****** * ***

**ForHIJ TTCCGCCTCGCCGAGTGCTTCTACGACCCGGGCACCGCCGCCGGCGCGGTCACCGCCGAC 399**

**GenHJ TTCAAGCTCCGCGAGACCTTCTACAGCGCGGTCACCGAGCCGGGCGACGTGTACGCCGAC 420**

***** *** **** ******* * *** ***** * **** ** *********

**ForHIJ GAGGTGGTGACGGGCTTCCTCGACACCCTCATCGGCGAACCGTCGCACATCGGCGGCCGC 459**

**GenHJ GAGATCAT------------------CTCCGGTGGCGAGGAGTCGCACGTATCGGGCCTG 462**

***** * * * * ***** ******* * ******

**ForHIJ ATCTTCTACACCGAGTTCATCGACGACCGCTGGCAGGAGGGCAAGCCCAACCGCGTCGTC 519**

**GenHJ ATCCACCACACCGAGTTCATCGACGACCGGTGGACGGACGGCACGCCCAAGCGGGTGCTC 522**

***** * ********************** *** *** **** ****** ** ** ****

**ForHIJ ACCGTCCTGGAGCCGCCGGCGCCCCCGGACAAGCCCGACGTGTCCAAGGTGGGCCGCACG 579**

**GenHJ ACCATCCTCCAGCCGGCCAAGCCGCCGGCCGAGCACGACCCCGAGAAGGTCGGCCGGGAC 582**

***** **** ***** * *** **** * *** **** ***** *******

**ForHIJ GCGCTGGAGGCGCTGGAGGGTCCGTCGACGTTCCAGTGCGCCGCGTCCGAGGTCATGGTG 639**

**GenHJ ATGGTCACCGTCCTCGACGGCCCGTCCACGTTCCAGGCCGGCGCCTCCGAGGTGCTGATC 642**

*** * * ** ** ** ***** ********* ** *** ******** ** ***

**ForHIJ CTCATGCGCCGCTACCTCGAGATGATTGAGCAGATGGACCTCGAGCGCGGTGTCACCCCG 699**

**GenHJ ATGCTGCGCCGCTACCTCGAGCTGATCGAGGAGATGGACATCGCCGCCGGCGTGACGCCG 702**

*** ***************** **** *** ******** *** *** ** ** *****

**ForHIJ CGGGTGCGCCTGTTCACCTCGCACCGGGTGGTGTCCACCGGCACCGACGAGGACGCCGGC 759**

**GenHJ CGGGTCCGGCTCTTCCTGTCGCACCGGGTCGCCGTCGGCGACGAGGGCGACGGTGACGGC 762**

******* ** ** *** *********** * * ** * * *** * * ******

**ForHIJ TACCTCAGCTGGCTCCGCCGCGAGGAAGGGTTCGTCAACTGCCCCGACGGGCGCAAGCGC 819**

**GenHJ TTCCTCAAGTGGATGCGGCAGGAGGACGGCTTCGTCAGCCTCCCGGACGGGCGCAAGCGC 822**

*** ***** *** * ** * ***** ** ******* * *** *****************

**ForHIJ ATCCAGATCGAGGCCGTGCGCGAGTTGGACTACAACGGCAAGTTCCGCCGGGTCCGCGTG 879**

**GenHJ CTCATCATCGAGGAGGTCCAGGAACTCGACTACCGGGGCCGGTACCGCCGGATCCGCAAG 882**

**** ******* ** * ** * ****** *** ** ******* ***** ***

**ForHIJ CCGGGTAGCAAGACCATCGACCTCGGCGTGCCGAAGCTGTTCATGATCGCCCAGGGGTTC 939**

**GenHJ CCCGGGACCCCGCTGATCGACCTGGGCGTCCCCGAGCTGTTCATGATCGCGCAGGGCTTC 942**

**** ** * * * ******** ***** ** **************** ***** *****

**ForHIJ AACAGCAGCGACGCGGAACGGCTCGGCTTCAAGCAGGAGGACGTCAAGGTCGACCACCAC 999**

**GenHJ GACAGCAGCGACGCGGACCGGCTCGGCTTCCGCCAGGAGGACGTCGCGGTCGACCACCAC 1002**

****************** ************ ************ ***************

**ForHIJ GACGGGCGTGGCGCCGTCGTCGCCCAGGCCGACTACCTCGCCGGGCTGCTGGAGATCCTC 1059**

**GenHJ GACGGCCGTGGAGCGGTCGTGGCCCAGGCCGACTACCTCGCGGGCTACCTCGAACTGCAC 1062**

******* ***** ** ***** ******************** ** ** ** * * ***

**ForHIJ GTCGACGGGCGGCTGCGCCGCCGCATCGCCTCCGACTTCGACAAGGAGGGCAACGAGTAC 1119**

**GenHJ GTCGGCGCCCGGCTCCGTCGCCGGATCGCCTCCGAGTTCCACCGCGAGGGCAACGAGTAC 1122**

****** ** ***** ** ***** *********** *** ** *****************

**ForHIJ TGGGTCCGCCAGATCGCCGTCGGCCACGAGGACGACGCCGAGGTCGGGTGGATCCTGGTG 1179**

**GenHJ TGGGTCCGCCAGATCGCGGTGGGCCACGAGGACGACCCCGAGGTGGGGTGGATCCTGGTC 1182**

******************* ** *************** ******* ****************

**ForHIJ CAGGTGCCCGACTACAAGACCTTCGACCCGATCCTCGCGGGGCTCGTGCCGCCCGGCACC 1239**

**GenHJ CAGGTGCCGGACTTCAAGACCTTCGACCCGATCCTGGCCGGGCTGGTCCCGCCGGGCACG 1242**

********** **** ********************* ** ***** ** ***** *******

**ForHIJ CACCGCAAGTCCAACGAATACCGGGCCGGCGTCCAGCACCTCATGCGCGAGTACTACCTG 1299**

**GenHJ CCGAAGCGGTCCAAGGAGTACCTGAACGCGTACCAGCAGCTGCTGCGAGAGTTCTACCTG 1302**

*** ****** ** **** * ** ****** ** **** **** *********

**ForHIJ GAGCAGGTATCGCTGATCACCGAGATGCCGGTCGCAGAGCTGGAGAAGGTCCAGTTGCCC 1359**

**GenHJ GAGCAGGTCTCGCTGATCACCGAGATCCCCGTCTCCGACCTGGAGAAGGTCCAGGCCCCG 1362**

********** ***************** ** *** * ** *************** ****

**ForHIJ TACGGGCCGAAGCTGTTCAGCCTCGTGGAGAAGGCCGGCGTCGACGCCCGCGTGGCGGTC 1419**

**GenHJ TACGGCCCGAAGCTGTTCAGCCTGATCGAGCGGATCGGCGCGGACGCGCTGGTCGCCGCG 1422**

******* ***************** * *** * ***** ***** * ** ** ***

**ForHIJ AACGGGGTCGTGGGCGGTGACACGTTCGGCAACGGGCATTTCCTCACCAGCGGCGGGGCG 1479**

**GenHJ AACGGCGTGGTGGGCGGGGACTCCTTCGGCAACGGCCACTTCATGACCAGCGGTGGCGCG 1482**

******* ** ******** *** * *********** ** *** * ******** ** *****

**ForHIJ ATTACCGGCATGATCGGGCACGCCTACCGGGTGCTGAAGTACTGGGAGGCGATCGACGCC 1539**

**GenHJ ATCACCGGCATGGTGGGCCACAGTTCCCGGGTGCTCCGCTACTGGCAGGCCCGCGACGCG 1542**

**** ********* * ** *** * ********* ****** **** ********

**ForHIJ GGCGTCGAGCACGACGTGGCCGTGCGCGAGCTCGCCGACGGCATCAAGCAGGACACGGAC 1599**

**GenHJ GGGGTCAGCCAGCAGCAGGCCATCCGTGAGCTGGCCGACCAGATCAAGGTGGACACCCTC 1602**

**** *** ** * **** * ** ***** ****** ****** ****** ***

**ForHIJ TGGTGGTTCCACGTCAGCGCCCAGGAGTTCAGCCAGGCCGTGCCGATCAACTTCGGCGCC 1659**

**GenHJ GGCTGGCTCCAGGTCAGCGCGCAGGAGTTCAGCCAGGCCGTGCCGATCAACTTCGGTGAG 1662**

*** *** **** ******** *********************************** ***

**ForHIJ GAGCGGATCGCCAAGATCGAGAAGGCGACCGGTCGTGACTCGGCGGCGCGCGCCAGCACC 1719**

**GenHJ GAGCGGATCAAGGAGATCGAGAAGCAGACCGGACGTTCCTCCGGCGAGCGGGCGAACACG 1722**

*********** *********** ****** *** *** * * *** ** * *****

**ForHIJ ATCGACGCCACCCGCCGGCACCGCCACTCGCTCGTCCCGCTGGACCCGTCGGACTGGCGA 1779**

**GenHJ ATCGACGCGACCCGGCGGCACCGGCACTCGCTCGTGCCGCTCGACCCGTCGGACTGGCGG 1782**

********** ***** ******** *********** ***** *******************

**ForHIJ CGGCTGCTGGTGCGCAGCGGCCGCATGCACGCCTTGGCGTTGCCGCCGATCCAGGACACC 1839**

**GenHJ CGACTCTCGGTGCGCAGTGGCCGACTGCACAGCAGCGAACTGCCGCCGCTCATGACGACC 1842**

**** ** ********* ***** ***** * * ******** ** * *****

**ForHIJ CACCCCGACCTGCGTGGTCCGGACATGATGCCGATGGACGCCAAGGCGA---------CC 1890**

**GenHJ CACCCGGCCGAACGGCAGATGATGCCCGACGACATGTCCGGAATGGGTGACGACATGCGG 1902**

******* * * ** * *** ** * ****

**ForHIJ GCCTGCATGGGTGGCGACGGCATGACGGGCAACGGCACGACGGGCAACGGCACGACGGGC 1950**

**GenHJ GAGACCGTCGCTGCCGGGGC-CGGTTCGGCGTCGTGACCAAGCTCAACCAACCCCGGGGT 1961**

*** * * * ** ** * * *** ** ** * * **** * *****

**ForHIJ GACGGGATGGCCGACGACGGCGCGAT------GCTGGCCGACA 1987**

**GenHJ GACTCGTACGGCGGCGGGGGAGAGACGGTCGTCCTGCGCGACA 2004**

***** * * ** ** ** * ** *** *******

**Fig. S1**. Alignment of the DNA segment coded for the *for*HIJ from the fortimicin biosynthetic gene cluster (NCBI accession code, AJ628421) with the DNA segment coded for *gen*HJ from the gentamicin biosynthetic gene cluster (NCBI accession code, AJ628149)

**GenS1CD2M2D1S2 -------------------------GTGACCCTTCTTGCCGTCAATGGTGGTTCCCCTAT 35**

**KanS1CD2M2D1S2 GGAGGTATGTGTCATGCCCCTGCAAAGTTCACGGCTTGCGGTCGACAACGGAACCCCCGT 60**

*** * ***** *** * ** **** ***

**GenS1CD2M2D1S2 ACGTTCGCAGCAGTGGCCGTTGTGGCCCGCTCCTGCGCCCGGCGCCCTCGACGCCCTGAA 95**

**KanS1CD2M2D1S2 CCGCGGCAAGCCCTGGCCGGTGTGGCCGCAGCCCACCGACGGCACCCTCGACGCCCTCTC 120**

**** *** ****** ******* ** * **** ***************

**GenS1CD2M2D1S2 TGAGGTTCTCCATTCCGGTCGGTGGGCCATCAGCGGGCCATATCAGGGGAAGCAGAGCTT 155**

**KanS1CD2M2D1S2 CCGCGTCCTGCGTTCCGGCCGCTGGGCCATCAGCGGCCCCTACCGGGGCGTGGAGTCGGC 180**

**** ** * ****** ** ************** ** ** * *** * ****

**GenS1CD2M2D1S2 CGAGCGGCGATTCGCAGCGGCGTTCGCCGAGTTCCACGAGATCGGGCACTGTGTGCCGAC 215**

**KanS1CD2M2D1S2 CGAACGCCGCTTCGCCCGCCGGTTCGCCGACTACCACCGCATCGCCCACTGCGTGCCCGC 240**

***** ** ** ***** ********* * **** **** ***** ***** ***

**GenS1CD2M2D1S2 CTCCAGCGGGACGGCCAGCCTCATGGTCGCGCTCGAGGCATGCGGGGTCGGTGCCGGTGA 275**

**KanS1CD2M2D1S2 CTCCAGCGGTACGGCGAGCCTGATGCTGGCCCTGGAGGCCTGTGGCGTCGGCGCGGGAGA 300**

*********** ***** ***** *** * ** ** ***** ** ** ***** ** ** ****

**GenS1CD2M2D1S2 CGAGGTTATCATCCCGGGCCTGACCTGGGTCGCCAACGCATCCACCGTCGCCGGGGTCAA 335**

**KanS1CD2M2D1S2 CGAGGTCATCCTGCCCGGCGTCACCTGGGTCGCCTCCGCCTCCACGGTGGTGGGCGTCAA 360**

******** *** * ** *** * ************ *** ***** ** * ** *******

**GenS1CD2M2D1S2 CGCGGTGCCCGTCCCGGTCGACGTGGATCCGCAGACCCTCTGTCTCGACCCGGCGGCCGT 395**

**KanS1CD2M2D1S2 CGCGGTGCCGGTGTTCGCCGACATCGACCCGGACACCCTCTGCCTCGACCCGGACGCCGT 420**

*********** ** * **** * ** *** * ******** ********** *******

**GenS1CD2M2D1S2 CGAGCGGGCGATCACCCCGCGAACCGCGGCCATCGTCGTGGTCCACCTCTACTCCGCCGT 455**

**KanS1CD2M2D1S2 CGAGGCGGCCATCACCCCGGCCACCAAGGCGATCGTCGTCGTCCACCTCTACGCGGCCGT 480**

****** *** ********* *** *** ******** ************ * *******

**GenS1CD2M2D1S2 GGCGGACCTGGACGCGTTGACCGCGATCGCCGAGCGGCACGAGATCCCGCTGATCGAGGA 515**

**KanS1CD2M2D1S2 CGCCGACCTCACCCGCCTCAAGGAGGTGGCCGACCGGCACGGCATCGTGCTCATCGAGGA 540**

**** ***** * * * * * * ***** ******* *** *** **********

**GenS1CD2M2D1S2 CTGCGCGCAGGCACACGGCGCGCGCTACCGCGACCGTCGGGTCGGCACGTTCGGCGCCTT 575**

**KanS1CD2M2D1S2 CTGCGCGCAGGCCCACGGCGCCGAGTTCGAAGGCCACAAGGTCGGCACCTTCGGCGCGGT 600**

************** ******** * * * ** ********* ******** ***

**GenS1CD2M2D1S2 CGGCACCTTCAGCATGCAGCACAGCAAGGTCCTGACCAGCGGTGAGGGCGGTGCCGTCAT 635**

**KanS1CD2M2D1S2 CGGCACCTTCAGCATGCAGCAGAGCAAGGTCCTGACCAGCGGCGAGGGCGGCGCCGCCAT 660**

*********************** ******************** ******** **** *****

**GenS1CD2M2D1S2 CACCGGGGACGCCGCACTCTCCCGGCGGGCCGAGCACCTGCGGGCGGACGGCCGCACCTA 695**

**KanS1CD2M2D1S2 CACCGCCGACCCGGTGCTCGCCCGCCGGATGGAACACCTGCGCGCGGACGGCCGCTGCTA 720**

******* *** * * *** **** *** ** ******** ************ *****

**GenS1CD2M2D1S2 CACGCCGGACGAGCCCGCCGTGGGAGAGATGGAACTGGCCCAGACCGCCGAGCTGATGGG 755**

**KanS1CD2M2D1S2 CCGCGATCAGGCGCCGCCCTCCGGCCACATGGAGCTCGTCGAGACGGGCGAGCTGATGGG 780**

*** * * *** ** ** * ***** ** * * **** * **************

**GenS1CD2M2D1S2 CAGCAACCGGTGCCTGTCGGAGTTCCAGGCGGCGCTCCTCCTCGGTCAGCTCGAACTTCT 815**

**KanS1CD2M2D1S2 CAGCAACCGCTGCATCTCCGAGTTCCAGGCAGCGGTCCTGACCGAGCAGCTGGGCGAACT 840**

*********** *** * ** *********** *** **** ** ***** * ****

**GenS1CD2M2D1S2 CGACGAGCAGAACGAACGGCGGCGGGCCAACGCGGCCCTGCTCGACGAGGGTCTCGGCGC 875**

**KanS1CD2M2D1S2 CGACCGGTTCAACGCCCTGCGACGGCACAACGCGGAACTCCTCGACGCGCTGCTGACCGA 900**

****** * **** * *** *** ******** ** ******* * ** ****

**GenS1CD2M2D1S2 GCTCGGCATCCAGCCCCAGGTCTCCTCGCCCGGCACCACCGAGCGCACCTACTACGAGTG 935**

**KanS1CD2M2D1S2 CGTCGGATACCGCCCGCAGCGCAGCACGCCCGGCACCACCGCCCGCACGTACTACACCTA 960**

****** ** ** *** * * *************** ***** ****** ***

**GenS1CD2M2D1S2 GGCCGGTCGGATCGAAGACGACGGGATCGGGCAGATCGGCGTCGAGCGGATCGCCCCGGC 995**

**KanS1CD2M2D1S2 CGTCGCCGAGCTGCCCGACGCGGAACTGCCCGGCGCGGACATCACCAAGGTCACCGAGGC 1020**

*** ** * * **** * * * * ** * ** ** *****

**GenS1CD2M2D1S2 GGTCGCCGCCGAACTCTCCGGCGCGGCCATCTACGCCAGCTACCCGCCGATGAACCACAA 1055**

**KanS1CD2M2D1S2 GCTGACCGCCGAACTCGGCTTCCCGGTGG---CACCGGCCTACTCGCCGCTCAACGCCAA 1077**

*** * *********** * * *** * **** ***** * *** *****

**GenS1CD2M2D1S2 CCGGCTCTACCAGCCCGCCACGCGGGCGCGGTTCAAGGGCATCGC------------CGG 1103**

**KanS1CD2M2D1S2 CCCCCTGTACGACCCGGCCAGTCGCAGCCGGTTCGCCCTCGGACCGCAGCACGAGAAGCT 1137**

**** ** *** * ** **** ** ****** * ***

**GenS1CD2M2D1S2 ACTCGACCTGACCGGCTACTCGTTGCCGGTCGCCGAGGACGCCGGCCAGCGGGTCGTCAC 1163**

**KanS1CD2M2D1S2 CATCGACCCCGCCCGATTCGTGCTCCCGGTGAGCGGCCGCCTGACGCGTCGGCTCGTCAC 1197**

******** ** * * * * * ***** ** * * *** *********

**GenS1CD2M2D1S2 CATCCACCACTCGGCGCTGCTCGGCGACGAGTCGGACATGAAGGACATCGTTCGCGCCTT 1223**

**KanS1CD2M2D1S2 CTTCCACCACGCCGCCCTGCTCGGCGACGAGTCGGACATGAGGGACATCGCGGAAGCGTT 1257**

*** ******** * ** ************************* ******** ** ****

**GenS1CD2M2D1S2 CGAGAAGGTGTTCGCGAATCACCGGGAACTACGCGGCTGACCACCGGGCCGGACGGGGAA 1283**

**KanS1CD2M2D1S2 CACCAAGGTGCTCCAGCACCGGGCCGTCCTGGCCGCTTGAGCCGAAGCCGTCACACACG- 1316**

*** ****** ** * * * * ** ** *** * * * ****

**GenS1CD2M2D1S2 GCTCCCCATCGACCCCGGCCCCGGCAGCTGCGATCGGGAGCCCCGACCCCGGGGTTTCCG 1343**

**KanS1CD2M2D1S2 ----------------------------------------------------CCTTCAGG 1324**

**** ***

**GenS1CD2M2D1S2 CTGGTTAGGGCCCCATCCAAGGGTTGTAGGGACCCGTGACGAAGGGTTGTGCGTATGGAG 1403**

**KanS1CD2M2D1S2 ATTGGGGACAGACCATG-------------------------------------CAGGTC 1347**

*** * **** ****

**GenS1CD2M2D1S2 GTCGAGATACGCCTGGGCTCGGTTCGGTATCCGTTCCGGCTCGGCACCGACTGCCTCGGT 1463**

**KanS1CD2M2D1S2 ACCACCATCACGATGGATGACGTCCAGTATCCCTACCGATTAGGCACGGACTGCCTCGAC 1407**

*** ** *** ** * ****** * *** * ***** ************

**GenS1CD2M2D1S2 GCCATCGTGGAAGACCTGGTCGCCATGTCGGCCAGTCGGCTCCTGATCGTCTGTGACAGC 1523**

**KanS1CD2M2D1S2 GGCATCGTCACGCGCCTCGGCGAACTCGGCGCCAGCCGCTACCTGATCGTCAGCGACCCC 1467**

*** ****** *** * ** * ***** ** ********** * *** ***

**GenS1CD2M2D1S2 AACACCGGTCCACTGTTCGGCGCGGAACTGGTCGAACGGCTCTCCCCACGGGT---TCCG 1580**

**KanS1CD2M2D1S2 AGGGTCGCCGAGCTGTACGGGCAGGGGCTGCGCGAACGGCTCGCGGAGCAGGCGGGACCC 1527**

*** ** **** *** ** *** ********** * * ** ****

**GenS1CD2M2D1S2 GCGAACCTGCTCATCCACCGCGCCGGTGAGCCGTACAAGGATCTACAGGCCGTCGGCACG 1640**

**KanS1CD2M2D1S2 GCCGAGCTGATCACCCATGCCTCGGGAGAACAGAACAAGGGCCTGCCCGCACTGCACGAC 1587**

**** * *** *** *** * * ** ** * * ****** ** * ** * ***

**GenS1CD2M2D1S2 TTGGCCGACTCGGCGCTCCAACTCGGCGCGGACCGCGCCTCGGTGGTGGTCGCCGTCGGC 1700**

**KanS1CD2M2D1S2 CTGGCCGAGGAGGCGCTGCGGCGCGGCGCCGACCGGCAGAGCATCGTCGTAGCACTCGGC 1647**

********* ****** * * ****** ***** * ** ** ** *******

**GenS1CD2M2D1S2 GGTGGCGTGATCGGCAACATCGCGGGCCTGATGGCCGCCCTGCTCTTCCGCGGGATCCGC 1760**

**KanS1CD2M2D1S2 GGCGGTGTCACCGGGAACATCGCGGGGCTGCTGGCCGCGCTGCTCTTCCGCGGCATCCGT 1707**

**** ** ** * *** *********** *** ******* ************** *******

**GenS1CD2M2D1S2 CTCGTGCACATCCCGACGTCGCTCATCGCGATGTCCGACTCGGTCCTCTCGCTCAAGCAG 1820**

**KanS1CD2M2D1S2 CTGGTGCACGTGCCCACCACCGTGGTGGCCATGCTGGATTCGGTGCTCTCGCTCAAGCAG 1767**

**** ****** * ** ** * * * ** *** ** ***** *****************

**GenS1CD2M2D1S2 GCGGTCAACGCCTGCGTGGGCAAGAACCTGATGGGGACCTTCTACGCGCCGGAGTCCGTC 1880**

**KanS1CD2M2D1S2 GCCGTGAACGCGGGAGTCGGCAAGAACCTGGTCGGCACCTTCTACCAGCCCGTCGAAGTG 1827**

**** ** ***** * ** ************ * ** ********* *** * ****

**GenS1CD2M2D1S2 CTGGCCGACACCGCCATGCTGCGGAGCCTGCCGTTCCGGGAGACGGTCTCCGGCCTCTGC 1940**

**KanS1CD2M2D1S2 CTCGCCGACACCGCGATGCTGCGCACCCTGCCGGTCCGCGAGGTCAGGTCGGGGATGTGC 1887**

**** *********** ******** * ******* **** *** ** ** * *****

**GenS1CD2M2D1S2 GAGGTCGTCAAGAACTCGCTGGCCATCCGGCCCAGCATGGTCGAGATGCTCCGCACGTCG 2000**

**KanS1CD2M2D1S2 GAGGTGGTGAAGAACTCGCTCGCCATCCGCCCCAGCATGATCGACCAGCTGTCGGCCGGG 1947**

******* ** *********** ******** ********* **** *** * ***

**GenS1CD2M2D1S2 CTGCGGCAGGACGCCGTCTACGACGACGAGACGATGTACGAGATCATCTCCGAGAGCATC 2060**

**KanS1CD2M2D1S2 CTGCGCCCCGACGGCCGCTATCCCGACGACACGATGCACTGGATCATCTACGAGAGCCTG 2007**

******* * **** * *** ****** ****** ** ******** ******* ***

**GenS1CD2M2D1S2 CTGGCGAAGGCCTCGGTGACCGTGGACGACATGCACGAGTGCCGCGCCGGTCTCGTGCTG 2120**

**KanS1CD2M2D1S2 GCCGCCAAGGCCCAGGTCACGGCGTACGACAAGTACGAGCGCGGCGAGGGACTCATCCTG 2067**

**** ****** *** ** * * ****** * ***** ** *** ** *** * *****

**GenS1CD2M2D1S2 GAGTACGGCCACACGGTCGGACACGCCATCGAGTACACCGCCGCCGGCGGGCTCTCCCAC 2180**

**KanS1CD2M2D1S2 GAGTACGGGCACACCGTCGGGCACGCCGTGGAGCACTCCTCGCAGGGAGCCGTGCCGCAC 2127**

********** ***** ***** ****** * *** ** ** * ** * * * *****

**GenS1CD2M2D1S2 GGCCAGGCGATCGGCCTCGGCATGGTGGTGGCCGCCGAGGTGTCCCACCGGCTGGGTCAC 2240**

**KanS1CD2M2D1S2 GGCGCCGCCGTCGCGCTCGGCATGATCGCCGCCGCCCAGGTCTCCCACCGGGCGGGCTGG 2187**

***** ** *** ********* * * ****** **** ********* *****

**GenS1CD2M2D1S2 CTCGACCAGGAGGCGGTCGCCCTGCACCGGGAGCTGTTGACCCGGGCGGGCGCGATGGTG 2300**

**KanS1CD2M2D1S2 GCCTCGGCCGAACTCGTCGACCTGCACCGGGAGCTCGTCGCCAAGACCGGGGTCGCGCGG 2247**

*** ** **** *************** * ** * * ** * * ***

**GenS1CD2M2D1S2 ACCATCCCCGAGGAGGTCGACCTCGACGAGGTGATGCACCGGCTGCGGTTCGACAACAAG 2360**

**KanS1CD2M2D1S2 CGCATCCCGTCCGACATACCGCTCTCCGCCGTCAGGCACCGCCTCTCCTTCGACAACAAG 2307**

******** ** * *** ** ** * ****** ** **************

**GenS1CD2M2D1S2 CGCGGCTACCTCGCCGACCCGGCCGAGAGCAGCGCGATGGTCCTCCTCGGTGGACTGGGC 2420**

**KanS1CD2M2D1S2 CGGGGCTACCTCCCGGCCTCCGCCGACACCTATCCGATGGTGCTGCTCGAATCCCCCGGC 2367**

**** ********* * * * * ***** * * ******* ** **** * *****

**GenS1CD2M2D1S2 GAGCCGCTGTGGCACGACGGGCGCCCGCTGGTCTCCGTTCCGATGGCGCTGGTCGGCGAG 2480**

**KanS1CD2M2D1S2 AAGGTGCTGCGCAGCGAGGGCACCGTCCTGACGGCGGCGCCACGGGACCTGGTCGACGCG 2427**

**** **** * *** ** * *** * * ** ** ******* ** ***

**GenS1CD2M2D1S2 GTCGTCAACGAGATCGCCCGTCCGGAGATCCCGAACTTCGAGTTGGTGGCTCCGGTCGAG 2540**

**KanS1CD2M2D1S2 GTGGTCGACGAACTCGCGGAACCCCCACGGCCCGCGGCCGCGAGGACC------------ 2475**

**** *** **** **** ** ** ** * ***

**GenS1CD2M2D1S2 ACGGTGGAGGAAGGCCGGGTGCCGGACACGGTGGGTGCTGCCGATGGTTGAGCGCCTGGG 2600**

**KanS1CD2M2D1S2 ---GACGACGCCG--------CCACCGTCCTCGGCGGTGCCGGGTGAGCGCCCCCGTGCG 2524**

*** ** * * ** * ** * * * ** * * * ** ***

**GenS1CD2M2D1S2 CGTCGCCGTAGTCGGCGGCGGGTTCATGGGTGGCGTGCACGCCGAGGTCCTGACCGCCGA 2660**

**KanS1CD2M2D1S2 CGTCGGCGTCGTCGGTGCGGGGTTCATGGGCGGGGTGCACGCCGAGGTGGTGGCGGCTCA 2584**

******* *** ***** * *********** ** ************** ** * ** ***

**GenS1CD2M2D1S2 TCCCCGAGTGGATCTGCGGTGGGTGGTGGACCGCGACGAGCGCGTCGGCACGGACCTGGC 2720**

**KanS1CD2M2D1S2 TCCCGGCGCCCGGCTCGAAGCGGTGCACGACCTCGACCCCGCCGCCGCCAGGGACCTGGC 2644**

****** * * ** **** **** **** ** ** ** ***********

**GenS1CD2M2D1S2 CACCCGGTTCGGCGCACGCGTCACCACGACCCTCGACGAGGC---GCTGGCTGACGACAC 2777**

**KanS1CD2M2D1S2 CGAGCGGTTCCGCGCCGAGCGGGCCGAGCCCTCCTGGGCGGACCTGCTCGCCGACCCCGC 2704**

*** ****** **** ** * ** * * ** *** ** *** * ***

**GenS1CD2M2D1S2 GGTCCGGTTCGTGGTGGTGGCCACTCCGGCCGCCACCCACGAGCCGATCGCGGCACAGGT 2837**

**KanS1CD2M2D1S2 GATCGACCTGCTCATCATCACCACGCCCAACGGGCTGCACCACCGGCAGGCGGCCGAGGC 2764**

*** ** * * * * **** ** ** *** * * * ***** *****

**GenS1CD2M2D1S2 CATCGCCGCCGGCCGGAACGTCCTGGTGGAGAAGCCGCTCGTGCTCTCCACCGGGCACGC 2897**

**KanS1CD2M2D1S2 GCTGCGGGCGGGCAAGCACGTACTGGTGGAGAAGCCGCTCGGTGTCACGCCGGAGCAGGT 2824**

*** ** *** * **** ******************* ** * * * *** ***

**GenS1CD2M2D1S2 CCGGCAACTCGCCGCCGCGGCCCACGAACGCGGGGTGGTCCTCGCGCACGGTGGCAACTT 2957**

**KanS1CD2M2D1S2 GGCCGAGCTCGTCGAACTCGCCGGACGGCACGACCGGGTCCTTGCCCACGGAAGCAACTT 2884**

*** **** ** *** * ** ****** ** ***** *********

**GenS1CD2M2D1S2 CGTCTACGCCCCGAAGTTCGTCCGGGCTCACGAGCTCGCGGCCGACCGGGAAGCACTGGG 3017**

**KanS1CD2M2D1S2 CGTGCACAGCCCGAAGTTCGTCCGGGCCCGTCAACTGGTCGCGGACACCGAGGCGTTCGG 2944**

***** ** ****************** * * ** * ** *** ** ** * ****

**GenS1CD2M2D1S2 CACCGTCCACTCCGTCCGGGTAGCCTTCCGGACGTCCGGACCGGACACCGACTGGTTCCG 3077**

**KanS1CD2M2D1S2 ACGGCCGCACCTGGTCCGGGTCGTCTTCCGCAACTCGGGCCCCGAGGCCGCCTGGGCCGC 3004**

***** ******** * ****** * ** ** ** ** *** **** ***

**GenS1CD2M2D1S2 GTCGAAGGCGACCGCCGGTGGTGGCGCCCTGACCGACCTCGGCTGGCACGCGGTCGAGCT 3137**

**KanS1CD2M2D1S2 GTCCAAGGACCTCGCGGGCGGCGGAGCCCTCCTGGACCTGGGCTGTCACGCGGTGGAGCT 3064**

***** **** *** ** ** ** ***** ***** ***** ******** *******

**GenS1CD2M2D1S2 GTGCCGCTGGATGCTCGGCAAGCCGGCCATCCGCGCGGTCACCGCCTGCA---------- 3187**

**KanS1CD2M2D1S2 GTGCCGGTGGCTGCTCGACGGCGCCGACGTCGAGTCGGTCAGCGCCCGACTGCAGCGGGT 3124**

******** *** ****** * * * * ** ****** **** ***

**GenS1CD2M2D1S2 --------------------------------------CCCGGCAGCTCAGTGCCGCCGG 3209**

**KanS1CD2M2D1S2 GCGGCCGCCCCACGACGCCGAAGCGGACCGCGCGTCCGGCACCGCGGGAACCGCGCGGGT 3184**

*** * * ** ***

**GenS1CD2M2D1S2 GGACGTCGAGGACCAGGGTGTCGTCCTGATCGAGTTCGCCGACGGCGCGATCGGTCAGTG 3269**

**KanS1CD2M2D1S2 CGCGCTGGAGGACCAGGCGCTGCTGGTCATGGAGTTCGCCGACGGCGCGGTCGGGCAGTG 3244**

*** * ********** * * * ** ****************** **** *******

**GenS1CD2M2D1S2 CGACGTCTCCTGGGCCTGCCCCGGCGGTGAGCAGCTCACCGTCGAGGTGATCGGCACGGA 3329**

**KanS1CD2M2D1S2 CGACGTCTCCTGGGTCACCCAGGGCGGTGAGCAGGTCACCGCGGAGATCATCGGCACCAA 3304**

**************** * ** ************ ****** *** * ******** ***

**GenS1CD2M2D1S2 GGGCCTGGTCACCGCCGATCTCTGGCAGGGCATGGGGGTCGAGGCCTACACCAACACCAA 3389**

**KanS1CD2M2D1S2 GGGCAGGGTCGAGGTCGACCTGTGGACCGGCATGGGGCTGCGCGCCTACTCGGACAAGGG 3364**

****** **** * *** ** *** ********* * ****** * *****

**GenS1CD2M2D1S2 GTTCGGCGCGGTGTGGGAGCCCAACCAGGGATGGCTGCGCCCCGAGTGGGAGTGGATCCG 3449**

**KanS1CD2M2D1S2 CTATCAGGACGTCTGGGATCCCGAGCAGGGCTGGGTGCATCCGGAATGGGAGTGGATCCG 3424**

*** * ** ***** *** * ***** *** *** ** ** ****************

**GenS1CD2M2D1S2 CAACAGCGGATACGTGCACCAGGACCGTCAGGTCGTGGACGCGGTGCTCGACGGGCGGCC 3509**

**KanS1CD2M2D1S2 GGCGAGCGGCTACTACCACCAGGACGGCACCGTGATCGAGGCGGTGGGCCAGGGCATCCC 3484**

******* *** ********* * ** * ** ****** * * ** ****

**GenS1CD2M2D1S2 C----------------------------------------------------------- 3510**

**KanS1CD2M2D1S2 CCTCACCCACGGCCCCGCGGAAGCGCTCGCCTCGGCCCGTGTCCTGGCCACCGGTTACCG 3544**

*****

**GenS1CD2M2D1S2 ------------------------------------------------------------ 3510**

**KanS1CD2M2D1S2 CAGTCACGCGGAGGGGCGGGTACTGCGGCTGTCCGGCGCGCCGGTCGGCCCTGGCGCGTC 3604**

**GenS1CD2M2D1S2 ---------------------ATGACGCACAC---------------------------- 3521**

**KanS1CD2M2D1S2 GACGACGGCGGCGGGCTCGGAATGACCGCCACCGAGCTCGGGGCGACCGCCGACACCGCC 3664**

******* *****

**GenS1CD2M2D1S2 -----------CCCGGACGACGCCGTCGCGGTCGTCGAAACG-CTC------------GA 3557**

**KanS1CD2M2D1S2 GGGCTCCGGAAGACCGCCGACGCCACCGGGCTCGGGATGACCGCCCGGGGCCGCGGCCGA 3724**

*** * ******* ** * *** ** * * ****

**GenS1CD2M2D1S2 AGCGGC--GTACCGCAGCGCGGCCGACGGGCGGAAA-GTGGAGATGAATGCCTGACAACA 3614**

**KanS1CD2M2D1S2 CCCGCCGCGGTGCCTCGTCCGTACCTCGTCCGCACCTCACCGATGGAGGGACCATGACCG 3784**

**** * * * * ** * ** ** * ** * * * ***

**GenS1CD2M2D1S2 AGGCCCCCCGGGGCGTCCTCTCGCTGACACCGTTCTTCCTCTACGCCGATCACCAGAAGC 3674**

**KanS1CD2M2D1S2 AGCCTGCCAAGGGTGTGCTCGCGCTGACTCCGTTCTTTCTGTACTCGGAGTACCAGGACC 3844**

**** * ** *** ** *** ******* ******** ** *** * ** ***** * ***

**GenS1CD2M2D1S2 TGTGGAAGCCACGGTTCGACCCGATGGGCGGCATGCACGTGTTGGGCCACGCGATCGTGA 3734**

**KanS1CD2M2D1S2 ACTGGGATCCCAAGTTCGACCCCATGGGCGGCATGCACCTGCTGGTGCGGGCCCTGGTCG 3904**

***** * ** ********* *************** ** *** * ** * ****

**GenS1CD2M2D1S2 CGGAGATGGGCCGGCGGGGCTTCCCGCAACGGGTCCTCACCATGGCCCCGCCCGGCGTCC 3794**

**KanS1CD2M2D1S2 AGGAGATGGCGGGCCGCGGCGTCCCGCACCGGGTCCTGACGATGTCACCGCCCAAGGTGC 3964**

********** * ** *** ******* ******** ** *** * ****** ** ***

**GenS1CD2M2D1S2 CGAAGGACCTCCAGATCGCCCCGAACGTCACCGTGCACGCCCGGCGACTGCCGGTGCTGC 3854**

**KanS1CD2M2D1S2 CCAAGGACATCAGGATCGGGCAGCGGATCAAGGTGCACGCCCGGCGGCTGCCCGTGCTGC 4024**

*** ****** ** ***** * * *** ************** ***** *********

**GenS1CD2M2D1S2 CGATCCCGTCCAAGCTGGAGGGTTACTTCGGCCTGGTCGGGGCATGGGCGAAGGCCAGCC 3914**

**KanS1CD2M2D1S2 CCATCCCCTCCGACCTGGAGGGGTACTTCGGACTCGTCGGCGCCTGGGCCAAGGGCAGCC 4084**

*** ***** *** * ******** ******** ** ***** ** ***** **** *******

**GenS1CD2M2D1S2 TGCTCTACGTGATGCGCAACAAGGAGCAGCTCAAGCGCGAGATCGGAATCGTGCACGCGC 3974**

**KanS1CD2M2D1S2 TGTTGTGGGTGCTGCGCAACCGCAAGCGGCTGCGCCGGGAGATCGGCGCGGTGCACGCGC 4144**

**** * * *** ******** *** *** ** ******** ************

**GenS1CD2M2D1S2 ACTGCGACGGTTCCGGATCCGCCCCGGCGTACGCGTACGCCGCGGCGCAGGTGCTCGACG 4034**

**KanS1CD2M2D1S2 ACTGCGACGGCTCGGGCGCCGCGGCCTTCTACCCGTATCTGATGTCCCGGATCCTCGGCG 4204**

************ ** ** **** * *** **** * * * * * **** ****

**GenS1CD2M2D1S2 TGCCGATCGTGTCGCACATCTACTCCTGCCGGTCGCTCACCCAGCACCCGACCACGGTGT 4094**

**KanS1CD2M2D1S2 TGCCGCTGGTGGTCCAGATCCACTCCAGCCGCTATCTGAGCCAGCACCCCACCACGCTCT 4264**

******* * *** ** *** ***** **** * ** * ********* ****** * ***

**GenS1CD2M2D1S2 TCGAGCGGGTGGTCGACCCGGTGGCGAAGTCGGCGGAGAAGTACGTCATCCAGCACTCCG 4154**

**KanS1CD2M2D1S2 TCGAGCGGGTGACCGACCCGATCGCCAAGTGGGCGGAGCGGCACGCCGTCCGCAAGGCCG 4324**

************* ******* * ** **** ******* * *** * *** * *****

**GenS1CD2M2D1S2 GCGCCGTGCTCACCCTCAGCGACAAGGTGCGCGAGCGGATCCGGGACGAACTGCACGTGC 4214**

**KanS1CD2M2D1S2 CCGCGGTCCTGATGCTCACCGACCGGGCCCGCGACGAGATGCGGCGCAAGGCCCAGCTGC 4384**

***** ** ** * **** **** ** ***** *** *** * * ** *****

**GenS1CD2M2D1S2 CGGACGACAAGGTGCACCGGCTGGCGCACCTGGTCACCGACAACTTCGTCGGGCACGACA 4274**

**KanS1CD2M2D1S2 CCGCGGAGCGCGTGCACCGGCTCGCGTACCTGGCCAGCGACCAGTTCAAGGACGCCGACA 4444**

*** * ** *********** *** ****** ** **** * *** * *******

**GenS1CD2M2D1S2 CCCCGGAGCGCCGGGAGGAACTGCGGCAGCGGTTCGGGCTGACCGACGACAAGCCGACCG 4334**

**KanS1CD2M2D1S2 CCGAGGCGAGGCGGGCGGAACTGCGGGAGCGCTACGGCCTC---GACGACCGCCCGATCG 4501**

**** ** * * **** ********** **** * *** ** ****** **** ****

**GenS1CD2M2D1S2 TCCTCTACGTCGGCCGGATCTCGTCCGAGAAGGGCGTGGACTGGTTCGTCAAGGCCGCCG 4394**

**KanS1CD2M2D1S2 TGCTCTACGTGGGGCGGATCGCCGCCGAGAAGGGCGTGGAGTACTACATCGAGGCCGCGG 4561**

*** ******** ** ****** * **************** * * * ** ******* ***

**GenS1CD2M2D1S2 CCGAGGTCG---CGAAGCGGCGCGACTGCCGGTTCCTGATCGCCGGGGACGGCCCACAGC 4451**

**KanS1CD2M2D1S2 CCGAACTGACCCGCAGGGGCCGGGACTGCCAGTTCGTCATCGCCGGGGACGGTCCCGCCC 4621**

****** * * * * ** ******* **** * ************** ** ***

**GenS1CD2M2D1S2 GCGGCGACATCGAGGCGCTGGCCCGCCAGCTCGGCGTCGCCGACAAGCTGGTCATCACCG 4511**

**KanS1CD2M2D1S2 GGCCGGACCTCGAAAAGCTGATCGGCGCCCGCGGGCTGCGCGACCGGGTGACCATCACGG 4681**

*** *** **** **** * ** * *** * **** * ** ****** ***

**GenS1CD2M2D1S2 GCTTCCTGCTCCCCGAGTACATCCCGTCGATCATCTCGCTCTCGACGCTGGCCGTCCTGC 4571**

**KanS1CD2M2D1S2 GCTTCATGTCCCACGAGTTCATCCCATCCATGATCTCCCTGGGCGAACTCGTGGTGCTGC 4741**

******* ** ** ***** ****** ** ** ***** ** ** * ** ******

**GenS1CD2M2D1S2 CCTCGCAGTACGAGGAACTGGGCGTCGTCGTCCTGGAGTACATGATGATGAAGCGCCCGG 4631**

**KanS1CD2M2D1S2 CGTCCCGGTACGAGGAGCTGGGCATCGTCATCCTGGAGTGCATGACCATGCGCAGGCCGC 4801**

*** ** * ********* ****** ***** ********* ***** *** * *****

**GenS1CD2M2D1S2 TCGTGGCGCACGACGTCAGCGGCGTCCACAAGCTGGTCGACCACATGAAGACCGGCGTCC 4691**

**KanS1CD2M2D1S2 TGGTCGCGCACGACGTGAACGGCGTCAACAAGCTCATCGAGGACGGCACCACCGGAATCG 4861**

*** ** *********** * ******* ******* **** ** * ***** ****

**GenS1CD2M2D1S2 TGGTGCCGCCCTTCGACCCTCCGAAGCTCGCCGATGCCATCGAGATGGTTCTCGACGATC 4751**

**KanS1CD2M2D1S2 TCGTACCCCCGTTCAGGACCCCGGAGATGGCCGACGCCGTCGAGCGGCTCCTCGACGATC 4921**

*** ** ** ** *** * *** ** * ***** *** ***** * * ************

**GenS1CD2M2D1S2 CGGACCTGGCCCGTCGCCTCGCGGAGAACGCCGAACCGATTCCCCAGCGGGAGTACTCGC 4811**

**KanS1CD2M2D1S2 CGGAGCTGCGCGAGCGGATGGCGGAGAACGCCGCCCCGCTGCCCGCCGCCAAGTACTCGC 4981**

****** *** * ** * ************* *** * *** ***********

**GenS1CD2M2D1S2 TGGCCTCGGCCGGTGAACGCCTCGAGGCGATCTACCTATCCCTCATGGAGGAGTCCTGAG 4871**

**KanS1CD2M2D1S2 TCTCCGCGGCGGGCGACCAACTGGCGGGCATCTACCGGGAGATCGGGCTGTGAGGGTCCT 5041**

*** ** **** ** ** * ** * ** ******* ** * * ***

**GenS1CD2M2D1S2 ATGACCGTC-ACTAACAAGATCGTCACCGGTGTCGCATTCCCACCGTCGTTGTTGGCGGA 4930**

**KanS1CD2M2D1S2 CCTCGTCTCCCCCCACCCCGACGACATCGCGCTGTCCTTCGGCGGCTGGGTCGCGGCCCA 5101**

**** * ** ** ** ** * * *** * * * *** ***

**GenS1CD2M2D1S2 GACCCCGCCGATCTCGGTGGCGACCCTCACGGCCTACCTTCGCGACAAGGGTATGCCGGC 4990**

**KanS1CD2M2D1S2 CGCCCGCGGGCTCGCGGCCAA---------GGGCTGG---CGGTTCGACCTGCTGACCGT 5149**

***** * ** *** ** ** ** * * ** * ***

**GenS1CD2M2D1S2 TGTCGGCCTGGACCTGAACGCGGACTTCAACGAGTACCTGCTCAACCGGGTCGAGATCGA 5050**

**KanS1CD2M2D1S2 CTTCGGCACGACCCTGTACGCGCCCCACAGCCCGCGCGCCGTCACCAAGGAGGCCATCAG 5209**

******* * **** ***** * ** * * * *** * ** * *****

**GenS1CD2M2D1S2 ACAGGTGCAGGGCCCGGAGAACACCCACGAGTTCACCAAGCCCTTCATCAAGCAGTTCTT 5110**

**KanS1CD2M2D1S2 CACGCTCCGGGAGCGCGAGGACCGCGACTACGC--------GCGCCGTCACGGG-CTGCG 5260**

*** * * ** * *** ** * ** * * * *** * ***

**GenS1CD2M2D1S2 CCTGAACCACATCACCGGGAACTACTTCACCGAGACGAACTTCGAGCAGTGGGACCTGCA 5170**

**KanS1CD2M2D1S2 GCTGACCTCTCTCCGGCAGGAGGACTGCTCCTGCCTGGGCATGGACGACGAG-------- 5312**

****** * ** * * *** * ** * * * ** * ***

**GenS1CD2M2D1S2 GCAGCAGTGCCAGGTGGCGCCGGAGAGCCTCTCCATCTGGGACCCGCCCTTCCCGTTCTC 5230**

**KanS1CD2M2D1S2 -GAGGAGCTCATCG--CCCCCGAGGCGACCGATCCTCGCCGGGCCGCCGTGCGGC----- 5364**

**** ** * * * *** * * * * ** * ***** * ***

**GenS1CD2M2D1S2 GTACTGTGAGTTCCTGTCGATCCTGCGCGACGAGCCGGAGCG---CGTCGCGAAGCTGGT 5287**

**KanS1CD2M2D1S2 --------AGCTGATCGCGGCGGCGCTCGCCGGGGCCGACCTGGTCGTCGCGCCGCTGGC 5416**

**** * * ** ** ** ** * * ** * ******* *******

**GenS1CD2M2D1S2 CCGGGACCCGGACG------CGAACATCTACCACGCCTTCTACCAGGAGAAGGTGGCCGG 5341**

**KanS1CD2M2D1S2 TGTCGGCGGCCACGTGGATCACCGCATCGTCCGCACCGCGGTGCGGCAGTCGCTCGGCGC 5476**

*** * *** **** ** * ** * * ** * * * ****

**GenS1CD2M2D1S2 CAAGGCCTCCGAGCTGGGCCTGATGGGCTTCTCGATCATGGGGTACAA------CCAGGT 5395**

**KanS1CD2M2D1S2 CACCCCCTG---CCTCTGGTACGAGGACCTGCCGTACGCGTTGGAGAGCCCCGTCGAGGT 5533**

**** *** ** * ** * * ** * * * * * * ******

**GenS1CD2M2D1S2 CATCC--CGGC----GCTGACCCTGGGCTACCTGATGAAGAAGGAGAACCCGGACC---T 5446**

**KanS1CD2M2D1S2 GCCCTCCGACCACCGGCCCTGGCTGGTCGACATCAGGGGGCACGAGGCGGCCAAGCGCGC 5593**

*** * ** **** * ** * * * * * *** * * ***

**GenS1CD2M2D1S2 CTACATCTGCTGGGGCGGCCCCTGGGTGACCTCCTTCGC---GGACATGCTCATCCCGCG 5503**

**KanS1CD2M2D1S2 CGATCTCGCGCTGTACCGCTCGCAGATGACCGCCGCCGACACCTCCGAGGTCCTCTCGT- 5652**

*** * ** * * ** * * ***** ** ** * * ** ** ****

**GenS1CD2M2D1S2 TCTGGAGGCCTGCCCCGAACTGGGTGAACTGATCGACGCCCTGGTCGTCCGGGAGGGCGA 5563**

**KanS1CD2M2D1S2 ---ACCGGCCGGACGGCGCGTCCGTCCCGTGCGAGCGGCTCTGGAGTTCG--------GC 5701**

****** * * * ** ** * ** **** ** ***

**GenS1CD2M2D1S2 GGAGCCGCTGCTGAAGATGGCCGAGGCGCTGTCCCGGGGCGAGCGGCCGGTCGGTATCCC 5623**

**KanS1CD2M2D1S2 GGGCTTCCCCCAGGACCTGGCCGAGCGGATGGCACTGGCCACGCTGGCCGCCG------- 5754**

**** * * * * ******** * ** * * ** * ** * * * ****

**GenS1CD2M2D1S2 GGCCCTCGCCGGGGCAGGTAGCCGCTGATGACGCAGAAACTGGCCATCCTCGGTGGCGAC 6883**

**KanS1CD2M2D1S2 -----TGACACCCGACAAGGAGTCGCTATGAGCAAGAAGCTGGCGCTGTTCGGCGGAACT 5809**

*** * * **** **** ***** * **** ****

**GenS1CD2M2D1S2 CCCGTCCGGACCCGGCCCTGGCCCGAGTGGCCGCACGTCGGCCCGGAGGACGTGGATCGG 6943**

**KanS1CD2M2D1S2 CCCGTACGGAACGAGGAGTTCTACGACGGCCCGCACATCGGGCCGCACGATCTCGACCGC 5869**

******* **** * * * *** * ****** **** *** * ** * ** ****

**GenS1CD2M2D1S2 CTGCGGACCGTCATCGAGAGCCGCAACCTCGGCGGCATCCCCTTCCCGAACACCATGCAC 7003**

**KanS1CD2M2D1S2 CTCAAATCCGTCCTCGACTCGGGGAACTTCGGCGGCATCCCCTTCCCCAACACCCACCAC 5929**

**** ***** **** * *** ******************* ****** *****

**GenS1CD2M2D1S2 CAGCAGTTCGCCGAGCGGTTCACCGCCAAGCTCGGGGCGAAGTACGGCCTCCTGGCGACC 7063**

**KanS1CD2M2D1S2 ACCGCCTTCGCCGACCTGTTCACCGGCAAGCTCGGCGCCCCGTACGGCCTGATGGTCTCC 5989**

********** * ******** ********* ** ********* *** ****

**GenS1CD2M2D1S2 AACGGCACGGTGACCCTCTCGATGGCGCTGCGCGCGCTCGGGATCCATGCCGGTGACGAG 7123**

**KanS1CD2M2D1S2 AACGGCACCATCAGTCTCTCCATCGCGCTGCGCGCCCTGGGCGTGCGTGCCGGGGACGAG 6049**

********** * * ***** ** *********** ** ** * * ****** ********

**GenS1CD2M2D1S2 GTGATCACCACCGCCTTCACCTGGGTCGGGACGGTCGCCGGCATCGTGCACGTCAACGCC 7183**

**KanS1CD2M2D1S2 GTGATCACCACCGGCTACACCTGGATGGGCACGGCCGCGGCGATCGTGCACATCAACGCC 6109**

*************** ** ******* * ** **** *** * ********* **********

**GenS1CD2M2D1S2 GTGCCGGTGCTGGCGGACATCTCCGACGACAACTGGTGCATCGACCCGGTGAAGGTCGAG 7243**

**KanS1CD2M2D1S2 GTCCCCGTCCTGGTGGACATCGACCCCACGACCTGGTGCATCGACCCGGCGGCGGTGGAG 6169**

**** ** ** **** ******* * * * ***************** * *** *****

**GenS1CD2M2D1S2 GAGGCGATCACCGACCGGACCAGGGCGATCATGGTGGTGCACCTGGGCAACCAGGTCGCC 7303**

**KanS1CD2M2D1S2 GCGGCGATCACCCCGCGCACCAAGGTGATCGTCCCGGTCCATCTGGGCAATCAGATCGCC 6229**

*** ********** ** **** ** **** * *** ** ******** *** *******

**GenS1CD2M2D1S2 GACATGGACGCGCTGCTGGACATCTGCCGCCGGCACAACCTGCTGCTCATCGAGGACTGC 7363**

**KanS1CD2M2D1S2 GACCTCGACGCGCTGCGGGCGATCGCCGACAAGCACGGACTCGCGATCCTGGAGGACACC 6289**

***** * ********** ** *** * * **** ** * ** * ****** ***

**GenS1CD2M2D1S2 GCGCACGCGCACTTCGCGGAGTGGCGGGGCCGGTGCGTCGGCACCATCGGCGACGCCGGC 7423**

**KanS1CD2M2D1S2 GCGCACGGGCACTTCGCCGAGTGGCGGGGGCAGTGCGTGGGCACCCACGGGGACGCGGGC 6349**

********* ********* *********** * ****** ****** *** ***** *****

**GenS1CD2M2D1S2 AGCTACAGCTTCGAGACCAGCAAGATCATGACCTCGGGCGAGGGTGGCTTCCTGGTCACC 7483**

**KanS1CD2M2D1S2 AGCTTCAGCTTCGAGAGCAGCAAGATCATGACTGCCGGTGAGGGCGGCTTCCTGGTGGCC 6409**

****** *********** *************** * ** ***** *********** ****

**GenS1CD2M2D1S2 GCAACCGAGGAGGCGTTCCACCGGGCGATGTCCCTGGCGCACGTCGGCCGCAAGGAGGCC 7543**

**KanS1CD2M2D1S2 AGGGACGAGGACGTGTACCAGCGGATGATGTCGCTGGCCAACTGCGGCCGCAAGGAGCCG 6469**

******** * ** *** *** ****** ***** ** ************* ***

**GenS1CD2M2D1S2 CCGTACGACAGGTTCCCGGGCCGGGTCTTCGGCTGGAACCACCGGGCCACCGAGATGCAG 7603**

**KanS1CD2M2D1S2 GGTTACGACGGTTTCGCGGGCCGCACCCTGGGCTGGAACGCCCGCGCGAGCGAACTGCAG 6529**

******** * *** ******* * * ********* *** ** * *** *******

**GenS1CD2M2D1S2 GCGGCCGTCCTGCTGGGGCAGCTCGACCGGTACGACGCCCTCGACAAGCAGCGGACCGCG 7663**

**KanS1CD2M2D1S2 GCCGCCTTCATGATCGGGCAGGTCGAGCAGCACGACGCGCTGCACGCCAAGCGGGCGGCG 6589**

**** *** ** ** * ****** **** * * ******* ** ** ***** * *****

**GenS1CD2M2D1S2 ATGGCGGAGATGCTGACCCAGGGGCTGGTCGAGATCGGCGGCTTCAAGCCGCTGGCGGAG 7723**

**KanS1CD2M2D1S2 AGCGCGGCGAAGCTCACCGCGGGGCTCGCCGAGATCGGCGGCTTCACTCCCGTGGGGAAC 6649**

*** **** ** *** *** ****** * ***************** ** *** * ***

**GenS1CD2M2D1S2 GAC---CCCCGGGTCACCCGCCGGCAGCGTTACGAGCTGCTCTTCCGGTTCGACACCGAG 7780**

**KanS1CD2M2D1S2 GACGACCCGAGGATCACCCGGCGCCAGTACTACGAGGTCATCTACCGCTTCGACCCCGCC 6709**

***** ** ** ******* ** *** ****** * *** *** ****** *****

**GenS1CD2M2D1S2 GCCTGGGACGGGCTCCACCGGGACAAGGTGCTGGAGGCGATCCTCGCCGAGGGCGTCGAG 7840**

**KanS1CD2M2D1S2 GCCTGGGAGGGGCTGCACCGGGACGAGGTCCTCTCGGCGATCCTTGCCGAGGGCATCGAG 6769**

********** ***** ********* **** ** ********* ********* *******

**GenS1CD2M2D1S2 TTCGAGGGGAACACCTTCTACCCGCCGATGCACCGCGACGAGCTGTTCCACATCACCGCC 7900**

**KanS1CD2M2D1S2 CTGGAGGGCGACGCCTTCTACCCGCCCGTACACAAGAGCGAACTGTTCGCGGTGGACGCC 6829**

*** ***** ** ************* * *** *** ****** * ******

**GenS1CD2M2D1S2 GACGACTGGCCGGCGATCCGGGAGCGCTACGGCGAGAAGATCGAGCCGGACGCGTTCCAC 7960**

**KanS1CD2M2D1S2 GTCCACTGGCCCATGATCGCCGAGAGGTACGGCGATCGGATCGGCCCGGACAGCGTCGAC 6889**

*** * ******* **** *** * ******** ***** ****** ** ****

**GenS1CD2M2D1S2 CTGCCGGTGGCGGAGCGGGTGGCCTTCGACGAAGCGGTGTGGATCCACCACTCCCTGCTG 8020**

**KanS1CD2M2D1S2 CTGCCCGTCGCCGACCGCGCCGCCGCCGACGAGTCCGTGTGGGTGCACCACGCGCTGCTC 6949**

******* ** ** ** ** * *** ****** * ****** * ****** * *******

**GenS1CD2M2D1S2 TCGGTGGAGCCGGAGGACGTACAGGACATGCTGGATGCGGTGGTCAAGGTCCGGGACAAT 8080**

**KanS1CD2M2D1S2 ACCGGCGACGACAAGGACCTCGGCGACATCCTCGAAGCCGTCGCCAAGGTCCGCGACAAC 7009**

*** * ** ***** * ***** ** ** ** ** * ********* *******

**GenS1CD2M2D1S2 CTGGGGGCGCTGAAGAAGAGCCTATGA 8107**

**KanS1CD2M2D1S2 CTGCGAGAACTGCACGACGCGAGCTGA 7036**

***** * * *** * * *****

**Fig. S2**. Alignment of the DNA segment coded for the *kan*S1D2M2D1S2 from the kanamycin biosynthetic gene cluster (NCBI accession code, AJ628422) with the DNA segment coded for *gen*S1D2M2D1S2 from the gentamicin biosynthetic gene cluster (NCBI accession code, AJ628149)

**Fig. S3.** Standard calibration curve of standard gentamicin (GEN) plotted as concentrations (µg/mL) versus Area Under the Curve (AUC) displayed by HPLC chromatograms (mAU)


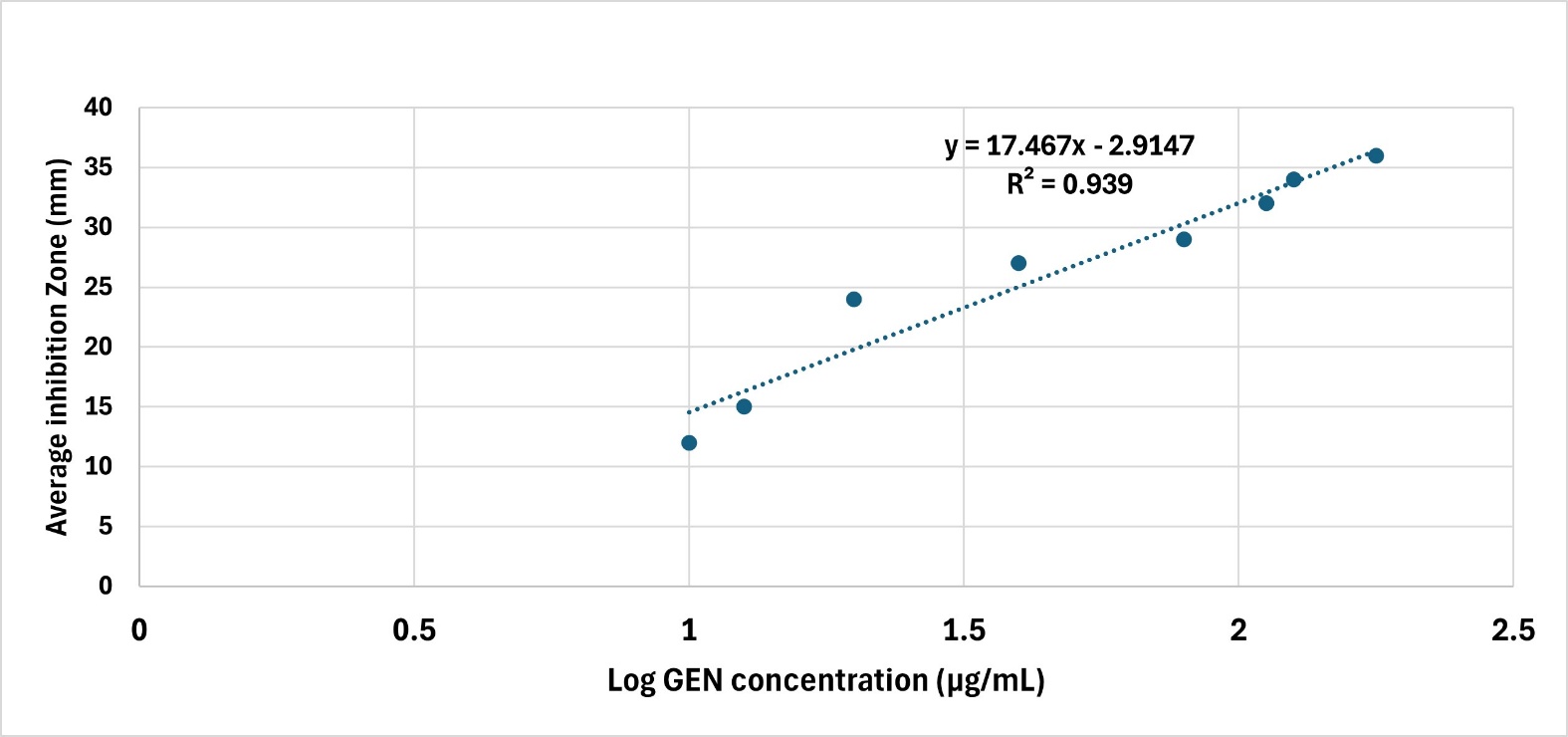


**Fig. S4**. Standard calibration curve of GEN created by plotting average inhibition zone (mm) versus log GEG concentration in µg/mL.


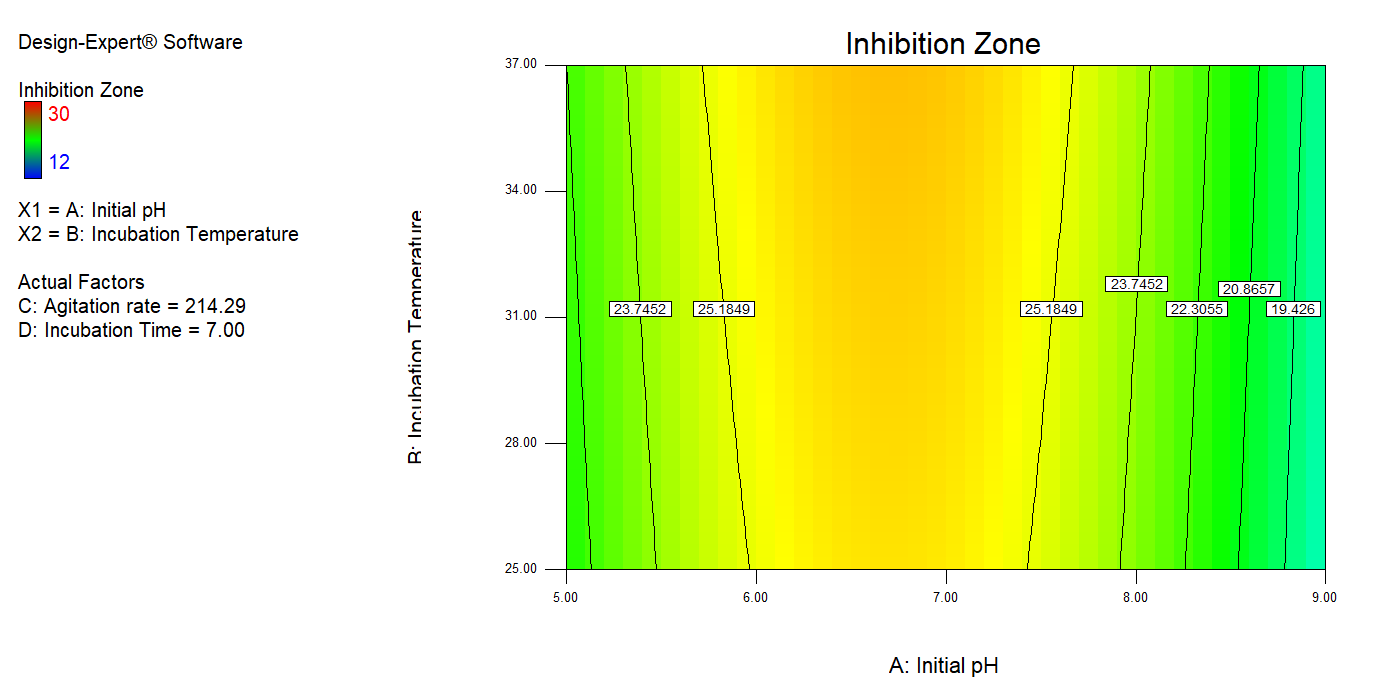


**Fig. S5**. Contour 2D illustrates the interaction of the two factors (initial pH, and incubation temperature) on the amount of gentamicin (GEN) produced by *M. purpureochromogenes* NRRL B-16094.


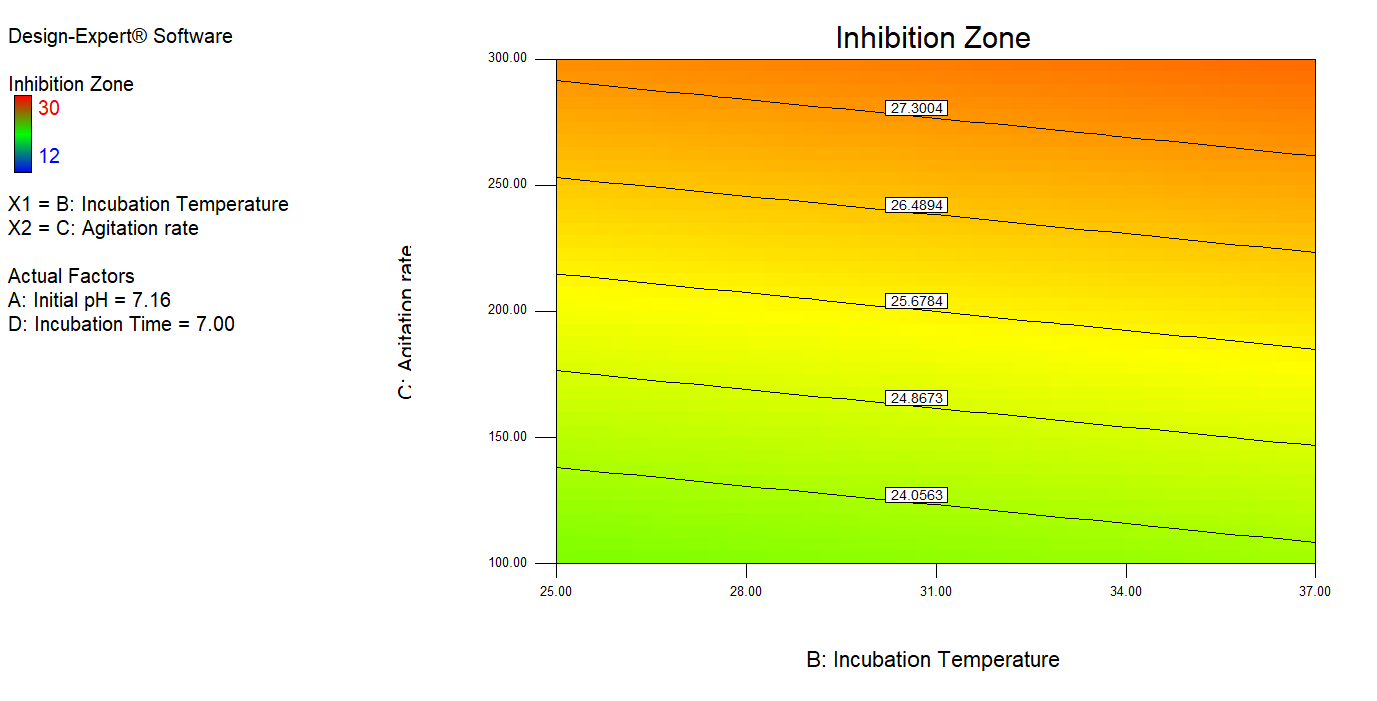


**Fig. S6**. Contour 2D illustrates the interaction of the two factors (incubation temperature and agitation rate) on the amount of gentamicin (GEN) produced by *M. purpureochromogenes* NRRL B-16094


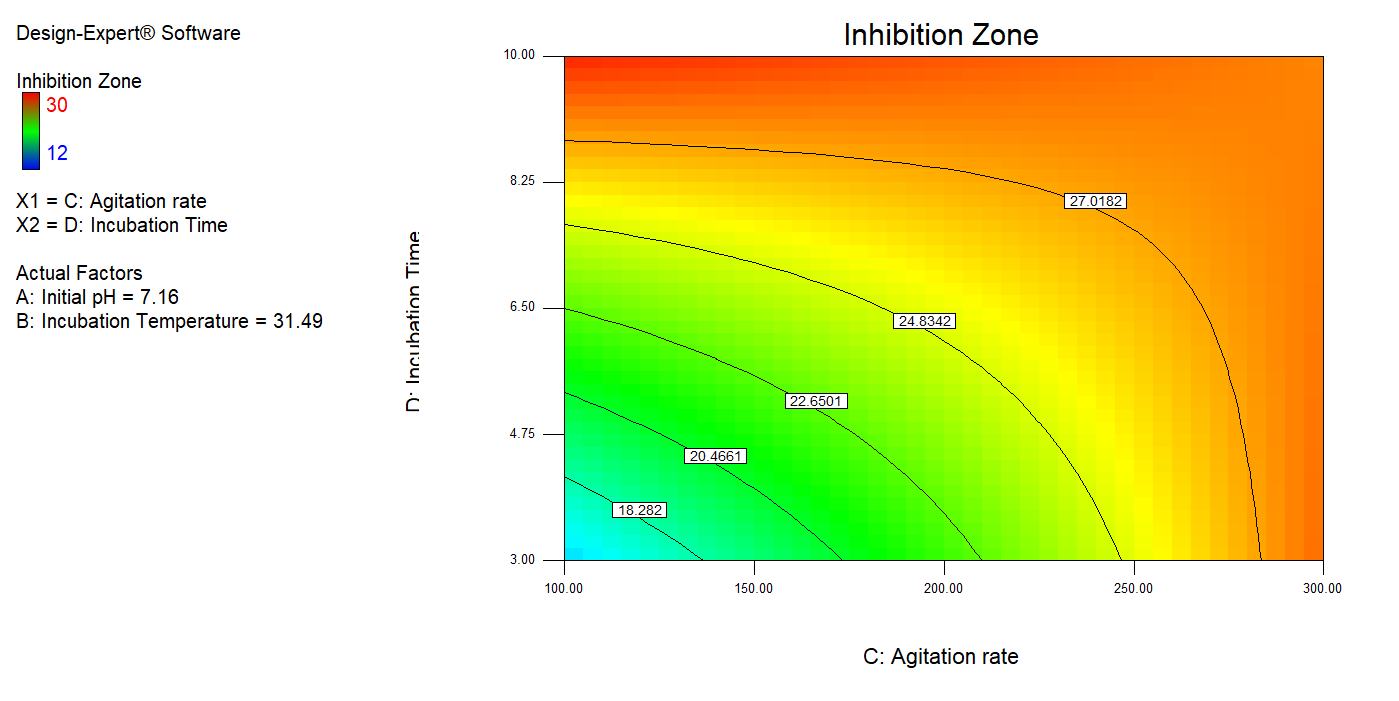


**Fig. S7**. Contour 2D illustrates the interaction of the two factors (agitation rate and incubation time) on the amount of gentamicin (GEN) produced by *M. purpureochromogenes* NRRL B-16094


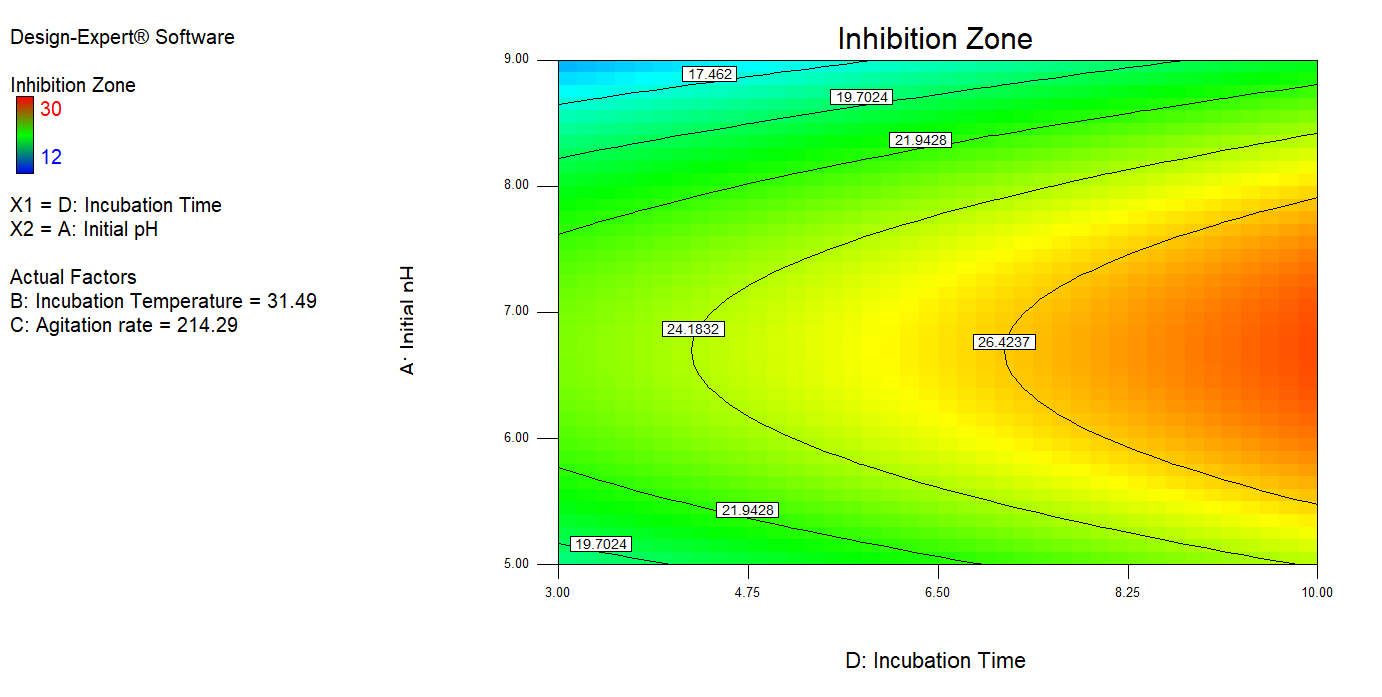


**Fig. S8**. Contour 2D illustrates the interaction of the two factors (incubation time, initial pH) on the amount of gentamicin (GEN) produced by *M. purpureochromogenes* NRRL B-16094


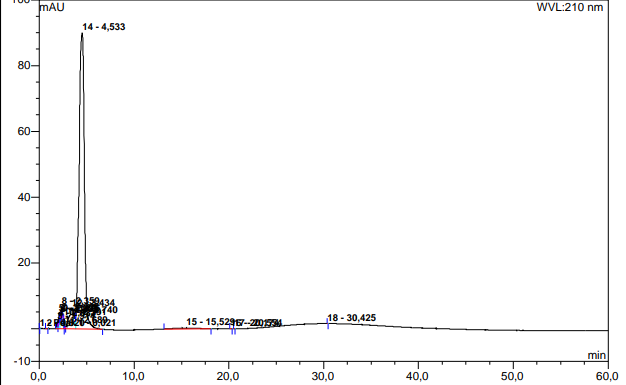


**(a)**


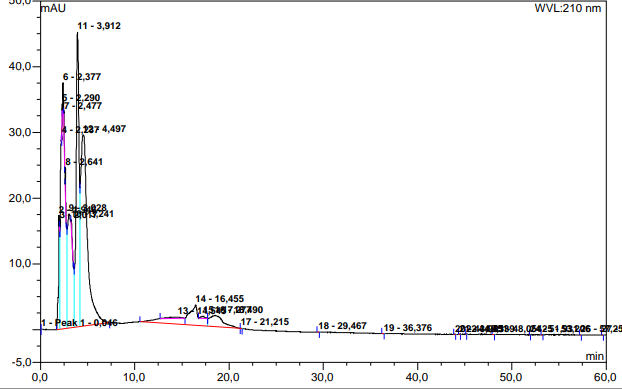


**(b)**

**Fig. S9.** HPLC chromatogram analysis of: (a) standard gentamicin (GEN) indicated by the arrow; (b) gentamicins (GENs) indicated by the red circle produced by *M. purpureochromogenes* NRRL B-16094.
